# Evaluation of the performance and achievements of the WHO Evidence-informed Policy Network (EVIPNet) Europe

**DOI:** 10.1186/s12961-020-00612-x

**Published:** 2020-09-24

**Authors:** Louise Lester, Michelle M. Haby, Evelina Chapman, Tanja Kuchenmüller

**Affiliations:** 1 Public Health, Nottinghamshire County Council, West Bridgford, Nottinghamshire, England UK; 2 Departamento de Ciencias Químico Biológicas, Universidad de Sonora, Hermosillo, Sonora Mexico; 3 Centre for Health Policy, Melbourne School of Population and Global Health, The University of Melbourne, Melbourne, Victoria Australia; 4 WHO Regional Office for Europe, Copenhagen, Denmark

**Keywords:** Knowledge translation, evidence-informed policy, network, evaluation

## Abstract

**Background:**

The Evidence-informed Policy Network (EVIPNet) is one of the key mechanisms introduced by WHO to reduce the research-to-policy gap. EVIPNet Europe was launched in 2012. We evaluated the performance and achievements of EVIPNet Europe with the overall aims (1) to inform future developments and strategic planning of EVIPNet Europe and (2) to contribute to the evidence base for organisational knowledge translation activities by sharing the lessons learnt.

**Methods:**

The evaluation covered the WHO Secretariat of EVIPNet Europe and its 21 member countries, from its inception to mid-2018. A mixed methods design was used to assess changes in three domains, including triangulation of quantitative and qualitative methods, based on the EVIPNet Europe Monitoring & Evaluation framework and theory of change. Data were collected between August and October 2018. Data collection comprised documentary review, social media analysis, online country evaluation, key informant interviews and validated tools. Two case studies were also developed.

**Results:**

The evaluation showed promising results as well as lessons to guide the future development of EVIPNet in the WHO European Region and other regions of the world. EVIPNet Europe appears to be filling a niche in promoting the capacity of Network member countries for evidence-informed policy-making. There is evidence that EVIPNet Europe’s capacity-building programme of work is improving knowledge and skills at the individual level. There has been an increase in activity and outputs since its establishment and evidence has been used to inform new policies in some member countries. However, the speed at which member countries are developing or publishing products varies greatly and no formalised knowledge translation platforms have yet been created. Financial and human resources are limited and staff turnover is a cause for concern, both at the WHO Secretariat and country team levels.

**Conclusions:**

Six years since the launch of EVIPNet Europe, the Network has grown quickly, is clearly valued and has had some successes. However, more work and support are needed if it is to achieve its vision of a Europe in which high-quality, context-sensitive evidence routinely informs health decision-making processes that ultimately serve to strengthen health outcomes across the Region.

## Background

Globally, it is known that translating research evidence into both policy and practice is a slow and often inefficient process [1, 2]*.* While more evidence now exists on how to translate the findings from research into healthcare practice, less evidence is available for the policy arena [1–4]. International commitment to evidence-informed policy-making (EIP) has increased over the past two decades as the use of robust evidence to inform public health policy is likely to ensure the greatest and most equitable population health gains [5], the greatest value for money in health services and health policy [6, 7], and greater transparency and accountability in decision-making [8].

Many barriers and facilitators for translating knowledge to improve EIP have been identified in a variety of settings and income levels [9]. Reviews suggest that some of the barriers are the lack of relevance and availability of research, including research that is locally applicable, no time or opportunity to use evidence, lack of funding, limited resources or high costs, lack of skilled policy-makers, insufficient institutional research capacity, frequent turnover of staff, inadequate dissemination of evidence, and a lack of priority on the policy agenda [10–13]. Conversely, the facilitators include improved dissemination of research, access to research, support of the administration, positive attitudes from managers, and close monitoring of the process and training of personnel [10–12]. Other facilitators include the quality of collaborations and relationships between research users and producers, the fostering of research co-production as well as research led by people embedded in the contexts in which the results can be used (e.g. policy-makers) [14–16].

Despite this increasingly popular field of research across the world, greater uptake and improved use of evidence within policy are required and still pose a challenge globally [12, 17, 18]*.* The methods or mechanisms used to close the gap between research and policy is known as knowledge translation (KT). Globally, there are many networks that promote KT activities, although these are often topic specific, e.g. Share-Net International [19] and the Joint Learning Network for Universal Health Coverage [20]. In this paper, we use the WHO definition for KT as: “*… the exchange, synthesis, and effective communication of reliable and relevant research results. The focus is on promoting interaction among the producers and users of research, removing the barriers to research use, and tailoring information to different target audiences so that effective interventions are used more widely*” [21].

The framework developed by Lavis et al. for assessing country-level efforts to link research to action is useful for considering the evidence of effectiveness of different KT activities to encourage the use of research evidence in policy [22]. Though targeted primarily at the country level, the authors acknowledge that some domains of the framework require a higher level of responsibility, e.g. production of systematic reviews is a global responsibility and global good. The four domains of the framework are general climate, production of research, a mix of activity clusters (push efforts, efforts to facilitate user pull, user–pull efforts and exchange efforts), and evaluation [22]. Of these domains, the majority of the interventions for which there is evidence of effectiveness have focused on the activity clusters but particularly on ‘push’, ‘facilitating pull’ and ‘pull’ activities [4]. Less is known about exchange or interventions to improve the general climate for research use and there are very few evaluations of efforts to link research to policy [22].

### The Evidence-informed Policy Network

The Evidence-informed Policy Network (EVIPNet) is one of the key mechanisms introduced by WHO to reduce the research-to-policy gap using a systems approach and to address the barriers to translating evidence, with the initial focus on low- or middle-income countries. EVIPNet is a global KT network established by WHO, with regional networks in development since 2005 [23]. EVIPNet’s mission is to promote a network of partnerships at the national, regional and global levels among health system policy-makers, researchers and civil society. Taken together, these are expected to strengthen health systems and improve health outcomes through regular access to and assessment, adaptation and use of context-specific research evidence [24].

EVIPNet (including EVIPNet Europe) is an organisational KT activity designed to improve the general climate for linking research to policy and to promote activities of linkage and exchange in order to institutionalise EIP [22, 25]. However, there is limited literature on the evaluation of organisational or system-level KT activities for EIP, with most evaluation activities concentrating on assessing individual-level changes [4, 26]. Previous attempts to evaluate KT and KT platforms (KTPs) in EIP include studies in specific EVIPNet member countries (e.g. in EVIPNet Africa, using case study [27] and structured reflection [28] approaches), focusing on evaluating specific activities (e.g. policy dialogue/evidence briefs for policy [29]) or looking at specific disease/topic areas (e.g. obesity [30] and international development [31]). However, evaluation has not previously been conducted of the processes, activities and effectiveness of EVIPNet Europe as a KT network of networks or of its individual country teams.

The overall aims of this formative evaluation were (1) to inform future developments and strategic planning of the Network and (2) to contribute to the evidence base for organisational KT activities by sharing the lessons learnt. Given the stage of development of the network, which is still in a growth and establishment phase, the evaluation focused on process and outputs, with only some short-term outcomes able to be measured.

### EVIPNet Europe

**Fig. 1:**
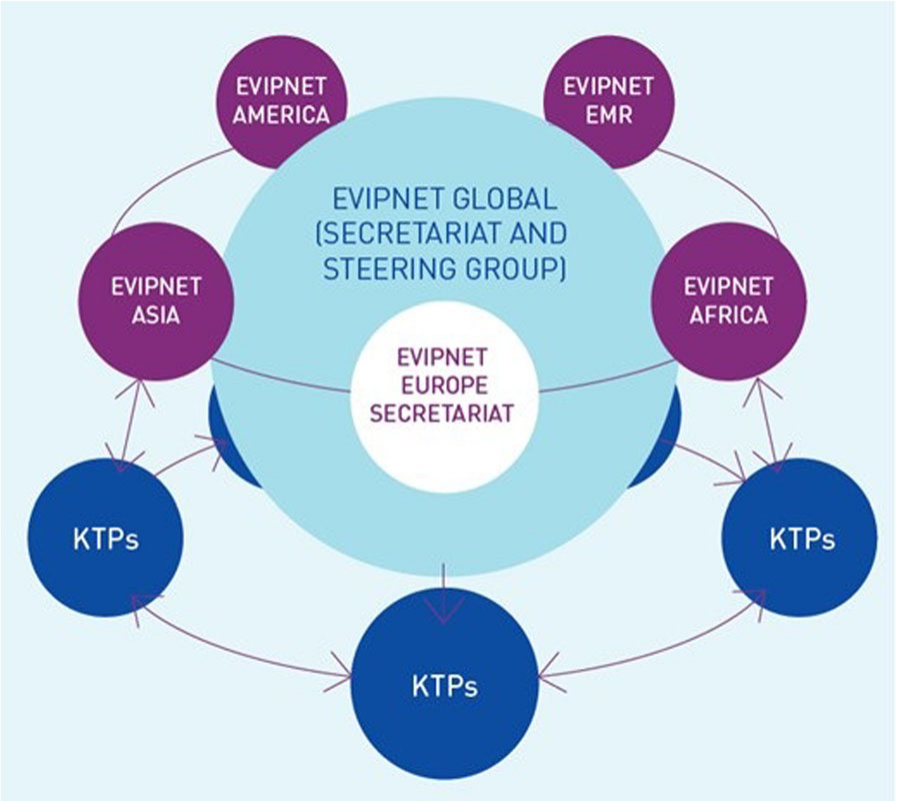
EVIPNet’s networked governance structure. Source: WHO Regional Office for Europe [25]

EVIPNet Europe, launched at the end of 2012, is a regional arm of the global WHO EVIPNet (Fig. 1). The vision of EVIPNet Europe is a Europe in which high-quality, context-sensitive evidence routinely informs health decision-making processes that ultimately serve to strengthen health outcomes across the Region [25].

As of mid-2018, 21 European countries had joined EVIPNet Europe. At a national level, individual country teams embark on a process to develop a KTP, which comprise key national actors (including policy-makers, researchers and civil society representatives) who lead the planning and implementation of KT activities and interventions. At the regional level, KTPs interact with each other to share experiences and lessons learnt, supported by the WHO Secretariat of EVIPNet Europe. As such, EVIPNet Europe functions as a centralized network of networks, i.e. the WHO Secretariat is at the hub and is connected with KTPs as network nodes.

EVIPNet Europe [25] links with a number of WHO strategic documents and initiatives, including the Sustainable Development Goals [32], the European policy framework Health 2020 [33], and the Action Plan to strengthen the use of evidence, information and research for policy-making in the WHO European Region [34]. EVIPNet Europe operates under the auspices of the WHO European Health Information Initiative [35].

### What were the strategic directions of EVIPNet Europe [25]?

*  Support KT networks*

EVIPNet Europe assists in the establishment of KTPs, which are national networks dedicated to strengthening innovative health partnerships among researchers, policy-makers and civil society in their respective countries in order to enhance EIP. These country-level KTPs are complemented wherever required and made feasible by the establishment and/or strengthening of regional and subnational networks.

*Strengthen KT capacity*

Recognising the limited capacity of KT in the region, EVIPNet Europe provides technical assistance, mentorships and exchanges, plus routine capacity-building workshops to improve the skill base of its network members.

*Support KT innovations*

EVIPNet Europe facilitates the development of KT strategies and tools tailored to the priorities of the countries in the WHO European Region.

*Catalyse KT at regional and national levels*

EVIPNet Europe promotes awareness and creates a commitment to improve the culture and practice of KT and EIP. EVIPNet Europe recognises that KTPs will be most successful and sustainable in regional and national environments that value the contribution of KT in health systems research and policy [25].

### What is a KTP and what does it do?

The KTP acts as an organisational knowledge broker between the research community, policy-makers (at different levels) and other stakeholders of a country [23, 36]. KTPs are a core element of EVIPNet Europe that are established in view of institutionalising the national EIP work. They launch and lead KT interventions at country level, including priority-setting exercises, evidence briefs for policy, policy dialogue exercises, rapid response services and capacity-building.

### Process to become a KTP

There are three steps that EVIPNet Europe foresees in developing a KTP [23, 36], as follows: Step 1 – undertake a ‘situation analysis’, which is a description and critical analysis of health policy, health research and practices of EIP in the country to assess the opportunities to establish a KTP. Step 2 – use the situation analysis results to construct three distinct KTP scenarios. Each scenario explores an organisational model for a KTP, including establishment considerations, possible policy-making activities, the organisational model’s strengths and weaknesses, and resource considerations. Stakeholders then convene to deliberate and assess each scenario against a set of criteria. Enriched by the tacit knowledge of stakeholders, the scenarios are submitted to the Ministry of Health and other key national stakeholders for final decision-making. Step 3 – The country team (or other designee) develops a formal strategy to operationalise the KTP, often through the creation of a strategic plan, business plan and/or operational plan.

The timeframe to go through this process to establish a KTP is very context specific, iterative and long term. A situation analysis (Step 1) is foreseen to be undertaken in 26 to 47 working days [37] but can take longer, depending on the country capacity. As for the implementation of all three of the above-mentioned steps, experience from other EVIPNet regions suggests that the establishment of a KTP is complex and can take up to 7 years [38, 39].

## Methods

**Fig. 2:**
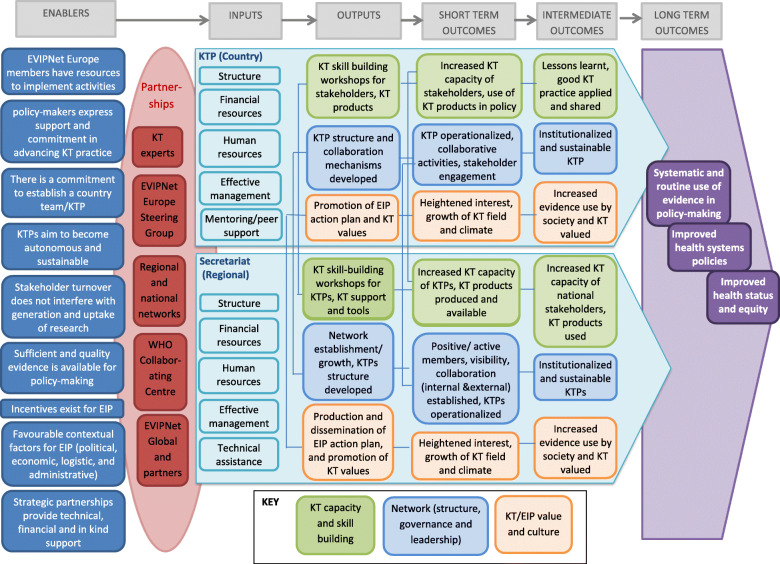
EVIPNet Europe Theory of change: research uptake and policies

The evaluation covered the WHO Secretariat of EVIPNet Europe and its 21 member countries from its inception at the end of 2012 until mid-2018. The evaluation was guided by both a theory of change (Fig. 2) that was developed as part of the EVIPNet Europe Monitoring & Evaluation (M&E) framework and the evaluation questions shown in Table 1. The evaluation measured changes in three domains that were identified and based on the strategic directions and cross-cutting approaches defined in the EVIPNet Europe Strategic Plan [25]. These domains include the Secretariat and the KTPs (countries) and are (1) KT capacity and skill building; (2) network structure, governance and leadership; and (3) KT and EIP value and culture (Fig. 2). A mixed methods design was used to assess changes in these domains and to answer the evaluation questions. Triangulation of both quantitative and qualitative methods allowed a thorough understanding of the nuances and context-dependent factors that influence the effectiveness of EVIPNet Europe at WHO Secretariat and country team levels. Given the stage of development of the network, which is still growing and establishing, the evaluation focused on process and outputs, with only some short-term outcomes able to be measured.

**Table 1:** EVIPNet Europe evaluation questions and sub-questions

Evaluation questions	Evaluation sub-questions
1. To what extent are the EVIPNet Europe objectives justified in relation to the current needs in countries? (Is EVIPNet Europe needed and accepted by member countries?)	a. How effective is the communication in both directions between the EVIPNet Europe Secretariat and the members of EVIPNet Europe? b. To what extent do member countries recognise and support EVIPNet Europe’s strategies, objectives, mandate and function? c. What factors at country level have facilitated or hindered the creation of stable and sustained research-to-policy activities and environments in EVIPNet Europe member countries?
2. Has EVIPNet Europe produced the expected results? (Is EVIPNet Europe achieving its objectives?)	d. How actively are EVIPNet Europe members engaged with the Network and participating in its activities at WHO Secretariat and country team levels? e. To what extent does EVIPNet Europe increase effective joint working, networking, collaboration and sharing of lessons between individuals and organisations at national, regional and international levels? f. What are the resources (human, financial, time and skills) invested in the work of EVIPNet Europe at WHO Secretariat and country team levels? g. To what extent have EVIPNet Europe and country teams developed skills and changed knowledge, attitudes and behaviours to use evidence in policy-making?
3. Does the development/implementation of EVIPNet Europe contribute to reaching higher-level objectives (such as KT and EIP)? (What are the outcomes of EVIPNet Europe?)	h. What are the accountability mechanisms for EVIPNet Europe (Secretariat and member countries) to measure the progress of EIP? i. To what extent have EVIPNet Europe’s portfolio of EIP tools been used by country teams to influence changes in policy? j. Does EVIPNet Europe fill a niche in the European health policy environment? k. To what extent do Network member countries value, promote and advocate for EVIPNet Europe’s function and EIP approaches throughout the WHO European Region? l. How have the relationships, contacts and exchanges created by EVIPNet Europe influenced the country EIP environment?

*EIP* evidence-informed policy-making, *EVIPNet* Evidence-Informed Policy Network, *KT* knowledge translation

Data were collected between August and October 2018 at both the WHO Secretariat and country levels. At the WHO Secretariat level, data collection comprised documentary review, social media analysis, online country evaluation and key informant interviews (KIIs). At the country level, documentary review, KIIs and validated tools were used. These are reported separately and were also developed into two case studies. Data collection and analysis were undertaken by an evaluator who was employed externally (LL).

### Quality assurance

In order to ensure that the evaluation met quality standards, the United Nations Evaluation Group and WHO evaluation guidance documents were used to develop the evaluation plan and final reports [40, 41]. These included addressing impartiality, independence, utility, quality, and transparency and integrating gender, equity and human rights in all aspects of the evaluation.

The M&E framework that guided the development of the evaluation plan was based on an extensive scoping review and externally reviewed by nine international experts before the evaluation started. An evaluation steering group was established to provide governance and technical input and oversight to the evaluation. In line with the participatory and co-production evaluation approaches used and proposed in other settings [42–44], stakeholders, including staff from the WHO Secretariat of EVIPNet Europe, national champions, WHO country office staff and external experts, were not just consulted but also actively involved in planning the evaluation.

### Sampling framework and selection criteria for countries

It was not possible to undertake a country-level evaluation with all 21 current EVIPNet Europe member countries. Further, many country teams are still in the early stages of development and thus certain indicators were not suitable for use for this formative evaluation. Therefore, a purposive sampling framework was used to identify two member countries to enrich the data collection via two in-depth case studies. This included the following criteria: geographical coverage, date of joining EVIPNet Europe and progress made to date. The aim of this selection was not so much that these countries are representative of all EVIPNet Europe member countries, given their context-dependent nature but rather that the two countries chosen (Republic of Moldova and Slovenia) are likely to be among the most advanced countries, with more lessons to learn from. Both countries joined EVIPNet Europe in 2013.

### Data collection methods

#### Documentary review

A review was conducted of existing publicly available and formal internal EVIPNet Europe-related documents from inception to September 2018. These were identified from the EVIPNet Europe website and internal files for the Secretariat level and by WHO country office staff for the country team level. The following documents were included: EVIPNet Europe multi-country meeting reports, annual reports, country team strategic and operational plans, technical documents/support instruments, KT products, minutes of EVIPNet Europe meetings, and policy and procedural documents. Documents available in English were reviewed.

#### Social media analysis

Online content was collated, comprising the numbers and types of discussions and documents shared as part of the closed Yammer® group for EVIPNet Europe members. It covered the period from inception of the Yammer group on 4 June 2015 to 13 July 2018. Data were collated by year for the main Network group only, and excluded the subgroups created.

#### Online country evaluation

An online evaluation was developed, piloted and circulated in September 2018 to all current 21 EVIPNet Europe member countries to assess a number of the high-level indicators at WHO Secretariat level and country team level. Invitations were sent to a member of the WHO country office and the national champion for EVIPNet Europe in all current 21 EVIPNet Europe member countries. The national champion is a trusted senior public health/health systems researcher or policy-maker or an intermediary (policy analyst or civil servant) who has experience in bringing the research and policy communities together. All answers were treated confidentially. The questions were piloted by two national champions from other regions of EVIPNet Global before distribution (questions available in Additional file 1, part A).

#### Key informant interviews (KIIs)

Semi-structured interviews were conducted with members of the WHO Secretariat, members of the EVIPNet Europe Steering Group, partners of the WHO Secretariat of EVIPNet Europe, and the two purposively selected country teams to explore their experiences of EVIPNet Europe. Purposive sampling was used to select participants for KIIs to provide rich and relevant data.

Interviews were conducted in English, either face to face or over the telephone/Skype during August–October 2018. Interviews were audio-recorded and then transcribed, alongside field notes made during and immediately after the call. The evaluation steering group reviewed the draft interview schedules, including prompts, to ensure that the main themes were included and to assess the appropriateness of the questions (Additional file 1, part B). The interview schedule was also piloted with a member of the WHO Secretariat before use.

#### Case studies

Case studies were developed for the two countries to detail their journey with EVIPNet Europe to date, including the challenges and successes as well as the lessons learnt. This allowed for a more in-depth exploration and understanding [45]. The case studies were drafted by the evaluator based on information gathered from the documentary review, KIIs and validated tools.

Three validated tools were applied in English in the two selected countries. These tools were the following:

*Is research working for you (IRWFY)?* This self-assessment tool looks at organizational, rather than individual, capacity to use research [46, 47]. National champions and WHO country office staff jointly completed seven multipart questions, scoring themselves from 1 to 5 across the areas of acquiring, assessing, adapting and applying research.

*Staff Assessment of enGagement with Evidence (SAGE).* The SAGE tool quantifies the extent of policy-makers’ research use based on an interview transcript and analysis of policy documents [48, 49]. The evaluator assigned SAGE scores based on interview data with a key policy-maker and the document review across the domains of research engagement actions and research use. The maximum score possible for each area is 9.

*Seeking, Engaging with and Evaluating Research (SEER).* National champions completed the SEER self-report tool consisting of 14 questions that assess the capacity to engage with and use research [48, 50].

### Data analysis

Descriptive statistics were tabulated to summarise quantitative indicators from the document review, social media analysis, online evaluation and validated tools (number, percentage, range, means, where applicable). Any missing data (e.g. if a question was not completed) were recorded as a separate category. The document analysis aimed to describe the context in which EVIPNet Europe, both at WHO Secretariat and at country levels, operates. This identified the range of network activities implemented and their influence on the EIP outputs and outcomes. Qualitative data from the KIIs and free text from the online evaluation were thematically analysed using content analysis. The data were systematically organised using an inductive framework approach [51] and adding themes that arose. There was a single coder only, but emerging themes were discussed with a member of the steering group. MS Excel® was used to manage all the data.

### Ethics

No formal ethical approvals were required as this evaluation was part of ongoing quality improvement. However, the evaluation was conducted in accordance with the principles outlined in the United Nations Evaluation Group Ethical guidelines for evaluation [52] and Norms and standards for evaluation [40]. Participants were invited to take part in the interviews on a voluntary basis, with consent obtained verbally at the start of the interview. Individuals were not identified in the outputs; anonymised quotes were used and content that identified individuals was censored. Countries will not be identified in external reports unless they provide consent.

## Results

Data were analysed from multiple sources (Table 2). Detailed results against each of the data collection methods are presented in Additional file 2 and the findings at the level of the two country teams are presented as case studies in Additional file 3. The case studies highlight the successes, challenges, KT capacity and skill-building, KT and EIP value and culture, lessons learnt, most significant change, next steps and resulting publications for each of the countries (Additional file 3). In addition, findings from all sources were used to address each of the evaluation questions and sub-questions (Additional file 4).

**Table 2:** Summary of data collected for WHO Secretariat and country-level evaluations

Method	Number of participants (participated/invited)	Date conducted
**Documentary review**	**42 documents**	
WHO Secretariat	30 documents	Aug–Sept 2018
Country team A	11 documents	Aug–Sept 2018
Country team B	11 documents	Aug–Sept 2018
**Social media analysis**	–	4 June 2015–13 July 2018
**Online country evaluation**	**23/40**	
WHO country office staff^a^	12/20	Sept–Oct 2018
National Champions^b^	11/20	Sept–Oct 2018
**Key informant interviews**	**16/19**	
WHO Secretariat	8/10	Aug–Sept 2018
Country team A	3/4	Aug–Sept 2018
Country team B	5/5	Aug–Oct 2018
**Validated tools**	**5/6**	
Country team A	3/3	Sept–Oct 2018
Country team B	2/3	Sept–Oct 2018

^a^ One EVIPNet Europe member country does not have a WHO country office

^b^ One EVIPNet Europe member country did not have an eligible national champion available at the time of the evaluation to invite

The countries that were members of EVIPNet Europe at the time of the evaluation are Albania, Austria, Bulgaria, Estonia, Georgia, Hungary, Kazakhstan, Kyrgyzstan, Lithuania, North Macedonia, Poland, Republic of Moldova, Romania, Russian Federation, Serbia, Slovakia, Slovenia, Tajikistan, Turkey, Turkmenistan and Ukraine. Of these, 13 countries joined in 2013, 6 in 2016 and 2 in 2018. Their progress as at the time of evaluation is represented in Table 3 by the type of EIP documents that they had produced since joining the network. Table 3 showcases only country-specific activities not the multi-country activities in which all the countries have participated.

**Table 3:** Progress of EVIPNet Europe member countries at the time of the evaluation

Country code and date of joining EVIPNet Europe	Progress made toward a KTP: Evidence-informed policy-making documents produced^**a**^
A. 2013	Situation analysis in development
B. 2013	Evidence brief for policy (2016) Situation analysis in development
C. 2013	Situation analysis developed and published in a peer-reviewed journal (2018) Evidence brief for policy, policy dialogue (2017), establishing KTP
D. 2013	EVIPNet Europe national launch workshop took place Situation analysis developed and deliberated at a stakeholder workshop (in the publication process) Evidence brief for policy in development
E. 2013	EVIPNet Europe national launch workshop took place Situation analysis in development Evidence brief for policy on hold
F. 2013	EVIPNet Europe national launch workshop took place Situation analysis and Evidence brief for policy in development
G. 2013	EVIPNet Europe national launch workshop took place Situation analysis in development Evidence brief for policy, policy dialogue (2017)
H. 2013	EVIPNet Europe national launch workshop took place Situation analysis in development Evidence brief for policy, policy dialogue (2017) Second evidence brief for policy in development
I. 2013	Rapid response report (2017)Evidence brief for policy in development
J. 2013	EVIPNet Europe national launch workshop took place Situation analysis (2017) Evidence brief for policy in development, establishing KTP
K. 2013	EVIPNet Europe national launch workshop took place Situation analysis developed and deliberated at a stakeholder workshop
L. 2013	Evidence brief for policy in development
M. 2013	
N. 2016	
O. 2016	Round table discussion (2017) Evidence brief for policy in development
P. 2016	
Q. 2016	EVIPNet Europe national launch workshop took place Situation analysis and Evidence brief for policy in development
R. 2016	Situation analysis and Evidence brief for policy in development
S. 2016	EVIPNet Europe national launch workshop took place Situation analysis initiated
T. 2018	Evidence brief for policy development initiated
U. 2018	

^a^ Note that only country-specific activities are included in the table. All of the countries will also have participated in multi-country meetings such as capacity-building workshops

*EVIPNet* Evidence-Informed Policy Network

### Financial and human resource inputs to EVIPNet Europe

EVIPNet Europe activities were mainly financed by WHO core funding, financing both the intercountry and the country-level work. Voluntary contributions were also obtained from the German Federal Ministry of Health for the years 2016–2018, amounting to US$ 170,000. Financial information available for EVIPNet Europe suggests that less than US$ 1 million has been spent by the EVIPNet Europe Secretariat between 2013 and mid-2018, which includes staff, equipment, travel and training for the network.

Financial resources were complemented by in-kind contributions in terms of interns, fellows and secondments working with the WHO Secretariat for a period between 3 and 6 months (such as from the Wellcome Trust through their Secondment Fellowship programme, the Carlo-Schmid Trainee Programme, the National Health Service England, or EVIPNet Europe national champions who worked for several months at the Regional Office to gain insights and contribute to the regional network activities). Furthermore, a range of partners such as the McMaster Health Forum, the Lebanon Knowledge to Policy (K2P) Centre and Cochrane as well as EVIPNet resource persons around the world, in particular the EVIPNet teams in Chile and Uganda, have provided their technical assistance to support the work of the network. Overall, in terms of staffing, the Secretariat consisted of the EVIPNet Europe coordinator supported, from mid-2014 onwards, by a full-time junior consultant.

### Themes identified across the evaluation methods

Analysis of the data from all sources identified a number of key themes. The main issues within each theme are presented in Table 4. More detail for each theme is presented below, illustrated using quotes from the interviews and analyses of the data from each source (Additional file 2), where relevant. These themes can be mapped against the three domains of the theory of change (Fig. 2). Domain 1 – KT capacity and skill building – is addressed in the network activities and processes theme; domain 2 – Network (structure, governance and leadership) – is addressed in three of the themes, including network growth, network activities and processes, and people; and domain 3 – KT/EIP value and culture – is addressed in the demonstrating value theme.

**Table 4:** Themes and sub-themes identified across data collection methods

**Demonstrating value**	**Network growth**
*Essential for building commitment to the Network and securing further resources and sustainability*	*EVIPNet has evolved over time both vertically and horizontally, which has created challenges*
• EVIPNet Europe is filling a gap – there are no similar networks in Europe at present • Gaining commitment is challenging because it is difficult to demonstrate value for evidence-informed policy • Sharing stories of success from other countries or regions helps improve understanding of, and commitment to, EIP • Further sharing of the EVIPNet Europe vision at the country level is needed • Greater use of strategic levers and advocacy to gather commitment at a high level will help gain commitment and provide a clearer mandate for countries to join the network	• Substantial horizontal growth, with the Network growing from 13 countries initially to 21 within a few years • The current resources are insufficient to fully meet member countries’ needs; integration into other programme-specific work and the use of BCAs^a^ to formalise the approach could help • The use of country cohorts^b^ to facilitate cross-WHO work is a promising approach • Large variability in development of Network member countries, making training and multi-country meetings more challenging to organise • There are challenges to the sustainability of country work, with many countries still requiring WHO funding and further technical support • There is a lack of institutionalisation (vertical growth), no KTPs are yet established • EVIPNet Europe has established a structure but many stakeholders are not aware of the accountability processes, e.g. through M&E
**Network activities and processes**	**People**
*The Network and tools created are valued and members feel they are useful and relevant* • The logical step-wise approach to, and methodology for, EIP are appreciated • The lack of a mandate (obligatory requirement) for EIP and associated products is a challenge for countries • The WHO Secretariat has developed five support instruments and resources to date, though some gaps for tools were highlighted • Technical capacity-building workshops and support instruments are an important part of the EVIPNet Europe approach and are valued, though more work is needed • Most communication is currently largely one way from the WHO Secretariat to member countries and the use of social network communication (Yammer) has decreased over time. Face-to face communication is important to establish and build relationships • Sharing and networking are important for sharing experiences and lessons learnt and for mentorship by other countries and regions	*Financial and human resources are limited and staff turnover both at the EVIPNet and country team levels is a cause for concern* • Individuals, personalities and relationships are important; WHO country offices play an important facilitating role; lack of coordination between agencies can be a barrier • Staff turnover at the level of the WHO Secretariat, WHO country offices, Ministry of Health, national champions and at the political level is a challenge • Partnerships with external and internal collaborators have been established at the Regional level; however, formal partnerships at the country level are more limited • Human capacity and financial resources were raised as an issue for country teams as well as the Secretariat; country teams are often reliant on limited staff, without dedicated funding or terms of reference • To help compensate for limited human and financial resources, the WHO Secretariat has been successful in utilising organisational resources, including core funding for intercountry work, and BCA funds (unique to the European Region) for country-level work^a^

*BCA* biennial collaborative agreement, *EIP* evidence-informed policy, *KTP* knowledge translation platforms, *M&E* monitoring and evaluation

^a^ The BCAs express an official commitment, as a contractual arrangement between the Member State and WHO to undertake EVIPNet Europe-related KT activities

^b^ The country cohorts are where a group of EVIPNet member countries work together on developing evidence briefs for policy for a particular issue (e.g. for antimicrobial resistance), supported by an external facilitator and complemented by guidance provided by the WHO Secretariat of EVIPNet and the programme team from WHO. This has allowed increased visibility and joint funding, increasing the resources available as well as inclusion in country BCAs and, therefore, within priority areas

### Demonstrating value

Demonstrating the value of the EVIPNet initiative is essential for building commitment to the Network, crystallising the vision and securing further resources and sustainability.

#### Filling a gap

Stakeholder and country interviews suggested a lack of awareness of any similar initiative currently existing in Europe. Most respondents of the online country evaluation (18/23, 78%) believed that their country had benefited from being part of EVIPNet Europe to date (Additional file 2; Fig. 2). However, there was a mixed response to increasing interactions between policy-makers, stakeholders and researchers (10/23, 43%), which requires more time for building trust, understanding and relationships.

#### Gaining commitment

Many stakeholders discussed the challenges of demonstrating the value of EVIPNet Europe. This was partially seen to be tied to perceptions that countries were already doing EIP, when in reality they were not, as evidence is used only in an ad-hoc manner or there are unrealistic ideals around what EIP can achieve, which are then not realised. This can manifest as countries thinking that they do not need EVIPNet. Others felt that there was a lack of understanding from senior policy-makers about what evidence is or that evidence is not used routinely even if the intentions are there. Furthermore, EIP was perceived as a complex and fairly abstract topic, for which success is not easily demonstrated and impact is not easily measured. This makes it challenging to demonstrate the return on investment and promote interest and long-term commitment.

“*I think a good approach has been to start with, for example, the evidence briefs for policy and then you can get people interested, you can show that this is a product that we can produce, and this is how it can be used and this is how it can make a change. People might understand better what this is about and might be more willing to commit to it.*” (Secretariat stakeholder, S3)

#### Sharing success

It is important to demonstrate the value of the Network’s activities through stories of success from other countries as part of EVIPNet Europe or globally to increase a country’s understanding and commitment to EVIPNet, particularly when it joins EVIPNet. In addition to the EVIPNet Europe introductory document Conceptual Background and Case Studies [23], collaboration between the WHO Secretariat and other EVIPNet Global member countries helps in sharing experiences and provides examples of good practice. Now that the Network is maturing, good examples and success stories of EVIPNet Europe are emerging (Additional file 3).

#### Sharing the EVIPNet Europe vision

The vision for EVIPNet Europe is frequently communicated through Secretariat-level products, including through its own strategy, evidenced in the documentary review. Lack of awareness of and engagement with EVIPNet Europe were mentioned as barriers at the country level by respondents to the online country evaluation, with suggestions that further promotion among the wider stakeholder community was required to support this national commitment. Using strategic levers and advocacy to gather commitment was felt to be an enabler for developing EVIPNet further, such as further discussion at high-level regional policies and meetings.

Secretariat stakeholders also expressed a need for greater awareness raising at a high level, providing a clearer mandate for countries to join the Network and demonstrating support for EVIPNet Europe as was done at the Sixty-sixth Regional Committee for Europe meeting, during which the EIP Action Plan was adopted, and at the Sixty-eighth Regional Committee for Europe meeting during the ministerial lunch on health information.

### Network growth

#### Horizontal growth

The Network has grown from 13 countries initially to 21 within a few years and more countries are seeking to join, especially western European countries. This demonstrates the need for EVIPNet Europe to increase country capacity in KT to support the implementation of the EIP Action Plan and catalyse the achievement of important societal goals such as the Sustainable Development Goals. This growth is also reflected in the social media analysis (Additional file 2, Part B).

#### Resources for growth

Growth of the Network presents both opportunities and challenges. Many said that the current resources were insufficient to fully meet member countries’ needs, with the Secretariat operating below the necessary staffing levels, often relying on a temporary outsourced workforce of consultants, interns, fellows and seconded persons. As this is likely to be exacerbated by further growth, there is a need for greater resources, both human and financial, to support an increase in Network members in order to maintain the current high standards and the existing close personal intra-Network relationships. Further, strategic decisions are needed on where the focus should be.

“*I think that the Secretariat is unfortunately understaffed, underfinanced and completely overworked.*” (Secretariat stakeholder, S3)

“*… because now we are broadening we are taking on board more and more countries and actually trying to engage them so actually we are spreading, and there is a demand for more countries, but we also need a strategy to support the countries who have subscribed in the beginning to maintain and continue their collaboration. So it’s actually a dual process.*” (Secretariat stakeholder, S2)

Another suggested area for improving Network growth and expanding support as well as income was working with other stakeholders and other WHO programmes, as many felt that the use of evidence was core to the work and mandate of WHO and important for all areas. Two promising approaches that were highlighted in the interviews are the country cohorts (Box 1) and biennial collaborative agreements (BCAs) (Box 2).

#### Variation in development

Another obvious challenge expressed was that this growth had led to an increased variability of Network member countries in terms of levels of awareness, skills and knowledge. Those who have been members since the beginning will clearly have different training and support needs to those that have just joined. Therefore, the training and multi-country meetings become more challenging to organise to meet the disparate demands of these members at different stages of development. More emphasis would be needed in the future on the use of the train-the-trainer model.

EVIPNet Global was identified as a possible source for the increased resources and global coordination required, including the unrealised potential for global capacity-building events.

“*I think what really is required is for us to have interaction with headquarters also in the capacity-building events* […] *that we have global events, global capacity-building events where these people can come together, that then depends on headquarters.*” (Secretariat stakeholder, S8)

#### Sustainability of country work

There are certainly sustainability challenges, which are likely to be exacerbated by further growth, with many expressing that, at this point, activities would not be possible or would not continue without WHO funding. There are initial signs of Network member countries having successfully submitted funding proposals and/or seeking their own funding. However, not all member countries seem to have the skills to write business cases and applications for funding. Additional training by the WHO Secretariat could be useful. However, technical training was considered a more important priority, as is securing stable staffing at country level to enable sustainability.

#### Vertical growth (institutionalisation)

Despite this growth, the establishment of KTPs within EVIPNet Europe is still required. Although there are examples of positive developments, these have yet to materialise formally. This lack of institutionalisation of KTPs was also expressed by many respondents during the country online evaluation. Many felt this was the obvious next step and area for future development, both strategically and in terms of support and capacity development for member countries. It was acknowledged that institutionalisation was not an easy or quick process but necessary if this Network was to become sustainable and self-sufficient.

“*So for me personally I think this is something that we have to tackle, because we are spending a lot of effort in the situation analysis … but for me personally not sufficient KTPs, concrete KTPs, have been established.*” (Secretariat stakeholder, S2)

#### Developing a structure

EVIPNet Europe has established a structure, including a Secretariat with dedicated staff to coordinate and support the Network. There is also a regional and global steering group and governance mechanisms. However, many stakeholders were not aware of the accountability processes for EVIPNet Europe and these have not been emphasised in publicly available documents. There was widespread understanding from stakeholder interviews that M&E was an important but challenging aspect to providing accountability. However, the documentary review revealed that no country evaluations have been conducted to date. It is expected that the development of the new M&E framework will create a new momentum, including the development of an additional simple data collection/reporting methodology. Progress is captured to some extent in the published annual reports.

### Network activities and processes

#### The EVIPNet approach

The logical step-wise approach to and methodology for EIP of the EVIPNet Europe process was appreciated. Interviewees and survey respondents highlighted the same standardised process to be followed, including how and where to look for evidence, considering EIP stakeholders, cost effectiveness, harms and benefits, and producing policy options, having a policy dialogue, etc.

“*… because you cannot disagree with the EVIPNet principles, it’s just so logical that things should be set up in this way.*” (Country team stakeholder, B1)

Despite the ability to adapt to the country context, stakeholders felt that the sequence in which countries develop KT products could be even further adapted to country-specific needs, such as starting off with rapid response mechanisms to provide opportunities for quicker wins and to demonstrate the benefits of EVIPNet Europe. This would also initiate the creation of a culture for EIP before embarking on concrete KT activities such as an evidence brief for policy and policy dialogue, if appropriate.

Challenges reported in the online country evaluation included a lack of available research evidence within countries or lack of awareness of the sources of evidence, the attitudes of policy-makers and lack of a culture of evaluation. The online country evaluation also highlighted the lack of mandate for developing evidence briefs as a challenge; while some countries followed the process, others needed a ministerial order.

#### Development of support instruments by the Secretariat

The documentary analysis highlighted five support instruments and resources developed and published by the WHO Secretariat to date [23, 37, 53–55]. Countries participating in the case studies reported using the support instruments and that they were relevant and useful, although many took longer than expected to finalise. However, the credibility of an official WHO publication was felt to be worth the trade-off in time and process, and important for maintaining the high technical standards and reputation to enable buy-in with senior decision-makers and other stakeholders. The policy dialogue process was particularly important in developing new relationships, exchanging opinions and expertise, and increasing understanding among experts and country stakeholders.

“*Yes, they were the basics for the work that we have done, so yeah, I mean I could speak for quite some time about each of them, but the general position is yes, each one of them has been very helpful.*” (Country team stakeholder, B4)

Using these EVIPNet support instruments, one situation analysis had been published [56] and seven were currently under development. In addition, two evidence briefs for policy had been published when the documentary analysis was conducted, with a further ten in draft form.

Some stakeholders thought that the development of further instruments might be overwhelming, while others provided examples of additional tools that could be developed, including more support for developing a rapid response service, instruments for appraisal of qualitative evidence, citizen engagement and organisational management (planned for the near future). One area mentioned by several stakeholders as a possible gap was more information or support for institutionalising a KTP within countries, including writing business cases, and how to finance a KTP, ensuring available resources to bring in topic experts while retaining a core of EIP experts.

#### Technical capacity-building

Capacity-development workshops are an important part of the EVIPNet Europe approach. The documentary review revealed that a number of face-to-face and webinar sessions have been conducted by the WHO Secretariat and have grown over time. This includes training as part of multi-country and national workshops. Despite no standardised evaluation question being used, average participant scores for these sessions are high. Interviews also highlighted the value of these capacity-building activities, in both the provision of technical skills and support for practical applications, not just knowledge, which were felt to be missing from other similar programmes. Mostly, it was changes in the skills and knowledge of participants that were reported, although some shifts in attitudes were suggested, particularly from the policy dialogues bringing different people together to understand each other.

Capacity-building support and the EVIPNet support instruments are clearly valued, with examples of how these had, or hopefully would, change policy. However, these currently benefit only those who attend sessions and/or develop country KT products. Some mentioned attempts at cascading this knowledge from those who had attended courses to stakeholders within countries but there was still a need identified to widen the pool of EIP experts within countries.

#### Intra-Network communication

Face-to-face communication between countries and with the Secretariat occurred mostly during the development of products and other specific country activities or during multi-country meetings. Most stakeholders suggested that communication was largely one-way from the WHO Secretariat, pushing information out and supporting countries. Others felt that some countries seemed somewhat uncomfortable in approaching the Secretariat for support, highlighting the need to build relationships and trust. Many stakeholders stressed the importance of face-to-face communication in establishing and building these relationships.

*“… but I think that the multi-country meetings are indispensable in the sense that if you don’t meet the person and see them and talk to them, then, I mean it’s easier to approach someone if you have met them in the past, so in that sense the multi-country meetings are a key moment in that communication.*” (Country team Stakeholder, B4)

Documentary analysis suggests that attendance at multi-country meetings has remained similar over time in terms of the numbers of attendees. However, attendance by WHO country office staff has reduced from 92% of member countries represented in 2013 to 16% in 2017. Some members and stakeholders also raised language issues in interviews and as part of the online evaluation. Despite translated products being available and training sessions being held with Russian interpreters, it was felt that this was not the same as having someone who truly understands EIP and the EVIPNet methodology and can explain and convey the details, nuances and passion accordingly, resulting in some elements being potentially lost in translation.

Social network communication across the EVIPNet Europe network, as indicated by the use of Yammer, has decreased over time and there has been variation in participation and the type of posts (Additional file 2, Part B).

#### Sharing and networking

The documentary analysis highlighted 18 conference presentations related to EVIPNet to date, and seven peer-reviewed publications authored by both the WHO Secretariat and country team members. Initially, these were mostly led by the Secretariat but, increasingly, countries are leading this external promotion, e.g. there are now four peer-reviewed papers with country members as lead authors. However, many of the papers have been published in WHO publications (*Panorama* and *Eurohealth*), with limited exposure to other sources, which may limit global reach, e.g. via journal database searches.

Sharing and networking were mentioned by several respondents of the online country evaluation, with lessons learnt and experiences exchanged with other countries cited as an important part of multi-country meetings, country cohorts, Yammer and direct country contact. The need for more opportunities for mentorship and participation in international events was suggested to enhance this further. Other examples included countries receiving mentoring from the other EVIPNet Europe member countries and EVIPNet Europe partners (e.g. EVIPNet Chile, K2P Center and McMaster Health Forum) and study visits to other countries as well as the WHO Regional Office for Europe to learn about EIP.

“[EVIPNet Europe] *is also a good networking platform and it supports us when I see other WHO* [country teams] *are struggling with the same difficulties and see how they have solved them, etc.*” (EVIPNet Europe national champion)

Greater involvement of EVIPNet Global and the role of headquarters in coordinating and ensuring knowledge exchange and sharing globally was requested.

### People

#### Relationships

Many of the interviewees felt that individuals, personalities and relationships were important. Individual members have developed strong relationships, with trust and rapport built and fostered over time by the dedicated Secretariat Coordinator, who is key to this. The role of individual personalities and networking skills was seen as vital to the success of the Network.

This related not only to the relationship with the Secretariat, with almost all representatives of Network member countries expressing gratitude for the support received, particularly from the Coordinator, but also the internal relationships with the WHO country office and Ministry of Health. Getting the right country team together was highlighted, with a need for team members to be highly motivated individuals. Within countries, developing a relationship between the national champion and WHO country office staff was an important part of the EVIPNet process, facilitated by WHO country offices. The latter are also crucial in coordinating and planning the future of EIP with the national champions.

In the country online evaluation, some countries reported coordination between agencies to be a barrier, e.g. interaction between policy-makers and the research community was lacking, coordination between ministries was slow, the Ministry of Health lacked a strong voice and had no interaction with the WHO country office.

#### Staff turnover

Different models were adopted for national champions, with some countries maintaining continuity where possible and others frequently nominating new representatives. While stakeholders recognised the pros and cons of these approaches (breadth versus depth), continuous retraining of champions and developing relationships as well as a lack of progression of knowledge and understanding caused by turnover of national champions were felt to be challenging. Furthermore, many stakeholders suggested that there was a need for countries to gain experience, not just in the technical skills-building capacity but also in applying this in practice, facilitated by hands-on technical support and coaching to develop understanding, as has been offered by the Secretariat. This is also made more challenging if staff turnover is high or untimely. Many references were also made to the challenges caused by turnover of staff in all areas, e.g. national champions, WHO country offices, Ministries of Health and the Secretariat. While there were examples of this turnover bringing about positive changes (e.g. shift in priorities to align with EVIPNet), this was mostly seen to be negatively impacting on Network development.

“*And in some countries they do nominate the same people for every year’s workshop so that they can, you know, increase skills; in other countries it’s more common that every year they want to give a different person the opportunity to participate so then it’s a bit difficult in terms of continuity, at the same time you’re exposing more people to the content.*” (Secretariat stakeholder, S3)

Staff turnover was also mentioned as causing delays to the creation of country KT products and institutionalisation of the process, with decision-maker/government changes being the main ones cited. Turnover at the political level also had implications for how members advocate for EVIPNet, given the need for political buy-in. However, with short terms of office, ministers want to see impact quickly, which is challenging, while changing policy and seeing a measurable impact takes longer, and M&E is also not yet well developed in member countries.

#### Creating partnerships

During the growth of the Network, the Secretariat sought and developed a number of collaborations, highlighted in the documentary review. These include external collaborations with HINARI, SORT IT (the Structured Operational Research and Training IniTiative), EUPHA (European Public Health Association), and the Cochrane Collaboration, K2P Center, McMaster Health Forum and Wellcome Trust. Internally, collaboration with the Division of Health Emergencies and Communicable Diseases has been established in the WHO Regional Office for Europe, which is expanding.

Formal partnerships at the country level in support of the work of national country teams at the time of the evaluation was more limited. However, there are indications of country teams undertaking intercountry collaborative activities, e.g. study visits, informal mentoring from EVIPNet Europe and EVIPNet Global member countries. Cohorts of countries working on the antimicrobial resistance evidence briefs for policy are a new collaborative step demonstrating added value as these have created communities of practice and allow sharing of experiences (Box 1).

#### Human and financial resources

Human capacity and financial resources were raised as an issue for country teams as well as the Secretariat. When asked about the human, time, skills and financial resources invested, some were not aware of these details. Of those who were, most suggested that resources were limited, and work was done by a small number of staff or consultants hired for a specific activity. Almost all finances to support EVIPNet Europe were from WHO. Country teams are often reliant on limited staff, without dedicated funding or terms of reference.

The WHO Secretariat has been creative in identifying alternative sources for human resources and expanded in-kind support, e.g. via half-year short-term fellowships and secondments. The document review highlighted the variation and turnover in WHO Secretariat staff and the variation in financial resources for the Secretariat over time. However, posts are included in the WHO human resource plans, highlighting commitment. Further, the operational plans suggest that the WHO Secretariat has continued to achieve its own EVIPNet Europe objectives, despite these limitations. The Secretariat has also been successful in utilising organisational resources, including core funding for intercountry work and BCA funds (unique to the European Region) for country-level work (Box 2).

Box 1 Country cohorts – encourages cross-WHO working A good example of cross-WHO working mentioned by several stakeholders was the country cohorts. A country cohort is where a group of EVIPNet Europe member countries work together on developing evidence briefs for policy, in this case on antimicrobial resistance-related briefs, supported by an external facilitator, complemented by guidance provided by the WHO Secretariat of EVIPNet Europe and the antimicrobial resistance team from WHO Europe. This has allowed increased visibility and joint funding, increasing the resources available as well as inclusion in country biennial collaborative agreements and, therefore, within priority areas. These cohorts have received promising feedback so far from stakeholders and member countries, including recognition from WHO headquarters and interest in replicating this model in other WHO regions.

“*… this cohort it’s about again instilling them with information, helping them prioritize the information that they really need to do the work and then working towards more of a Community of Practice model where there’s shared agreement on the deliverables each month, and people pre-circulate documents.*”

(Secretariat stakeholder, S5)

Box 2 Use of BCAs – bringing in other sources of funding The Secretariat has also been successful in utilising organisational resources, including core funding for intercountry work, and biennial collaborative agreement (BCA) funds (unique to the European Region) for country-level work. The BCAs express an official commitment as a contractual arrangement between the Member State and WHO to undertake EVIPNet Europe-related knowledge translation activities.

BCAs are useful for formalising country-level work with other WHO programme areas. These contractual elements with member countries and internally within WHO were also highlighted as an accountability mechanism by stakeholders and further use of BCAs is encouraged.

“*I think there’s a big benefit in liaising with other programmes and sort of offer a package to a country.*” (Secretariat stakeholder, S7)

## Discussion

### Key messages

- EVIPNet Europe appears to be filling a niche in promoting the capacity of Network member countries for EIP, with the recent growth of EVIPNet Europe demonstrating the demand for such an initiative in the Region.
- The Network and its activities are valued and EVIPNet Europe’s tools and approaches are perceived as useful overall. Furthermore, the Network’s capacity-building efforts have improved KT knowledge and skills at the individual level.
- The EVIPNet Europe approach has catalysed the planning and implementation of country-led KT activities and led to conceptual and instrumental policy influences.
- However, not all member countries are progressing at the same pace, demonstrating that a flexible and tailored approach and timing are important for success.
- Whilst finance is needed to ensure progress and sustainability, people and relationships are just as crucial to developing a culture of EIP in member countries and across the EVIPNet Europe network.

This formative evaluation showed promising results as well as lessons to guide the future development of EVIPNet in the WHO European Region and other parts of the world. The evaluation showed that a strong Secretariat is essential in setting up a KT network to strengthen the KT capacity of, and collaborative relationships between, Network members. While no country has gone beyond the formation of country teams, many countries have developed situation analyses and are in the process of paving the way towards creating sustainable KTP structures. Commitment and engagement from Network members vary with financial and human resources, including staff turnover, which are issues both at the EVIPNet Secretariat and country team levels.

Alongside its capacity-building efforts, the WHO Secretariat put in place numerous and diverse mechanisms early on to develop joint working, exchange of experiences and networking. These included peer support accelerated by the Network’s train-the-trainers programme, mentoring (within and beyond EVIPNet Europe member countries), and the face-to-face multi-country meetings and workshops. These mechanisms and the need for networks to have face-to-face interaction in building relationships on which knowledge that was co-produced can then be shared was highly valued by Network members, and has been recognised by others [11, 57]. Lake et al. suggest that “*it is perhaps not the information-passing that is the active ingredient in sharing knowledge but the trust, increasingly shared language, and learning about different perspectives, not achievable electronically, that make the difference*” [57].

The work of EVIPNet is legitimised and reinforced by being grounded in the resolution on health research of the Fifty-eighth Meeting of the World Health Assembly [58] and is supportive of the WHO Thirteenth General Programme of Work 2019–2023 [59] and the achievement of the Sustainable Development Goals [32]. In the European Region, EVIPNet Europe is an integral component of the WHO Europe Action Plan and resolution to strengthen the use of evidence, information and research for policy-making in the WHO European Region [34]. This strategic and policy embedment provides the Network with an official mandate for its work. It encourages member countries and holds them accountable to increase their investments in KT, including the establishment of KTPs in line with EVIPNet Europe’s aim to foster the systematic use of information in decision-making [34], which would in turn provide a country-level mandate for EIP and a greater likelihood of sustainability [60].

Furthermore, the work of EVIPNet Europe (and EVIPNet Global) is facilitated by the fact that WHO is seen as a credible and neutral broker and is an evidence-guided, normative organisation. Thus, it is in a privileged position to take the lead to support member countries to institutionalise EIP. The institutionalisation of EIP will require multiple and concerted efforts and high-level commitment from all players – at country level from policy-makers, researchers and the civil society and across all levels of the EVIPNet network, including the EVIPNet Europe Secretariat, WHO country offices and country teams/member countries [28, 60–62]. Further, as shown in previous evaluations, for KTPs to have the greatest chance of success and institutionalisation, it will be important to ensure a clear mandate, strong leadership, and sufficient funding and human resources [28, 60, 62, 63].

The results of the evaluation reflect that this is a formative evaluation; the financial and human resources available to support the Network are limited; the Network is rather large and has grown quickly; and that capacity-building takes time. Financial information available for EVIPNet Europe suggests that less than US$ 1 million has been spent between 2013 and mid-2018, which suggests that it is offering value for money when compared to other programmes. For example, the Building Capacity to Use Research Evidence (BCURE) programme received £15.7 million (approximately US$ 19.3 million) funding from 2013 to 2017 to build capacity to improve the use of evidence in decision-making in low- and middle-income countries through six linked projects implemented across 12 countries in Africa and Asia [31]. Similarly, an Australia-wide KTP focused only on community-based initiatives for obesity prevention received 3.4 million Australian dollars (approximately US$ 2.4 million) over 3 years (from 2013 to 2015) [30] but has since been discontinued.

This formative evaluation of EVIPNet Europe identified the strengths and good practices that should be further built upon. For example, the country cohort models for evidence briefs for policy (e.g. as used for antimicrobial resistance) enabled the integration with WHO programme-specific work as well as access to the generous funding that antimicrobial resistance is currently receiving from the donor community. The country cohort models also created communities of practice, an approach that is seen as promising in the KT community [64–66]. The targeted funding through the country BCAs (unique to the WHO European Region) is a comparative advantage of this Region and allowed the formalisation of country-level EIP work with other WHO programme areas. Other strengths/advantages of the European Region include WHO country office engagement with EVIPNet Europe and within countries; EVIPNet Secretariat’s dedicated funding of core staff to coordinate and support the Network, which is essential for Network establishment; multi-country meetings providing a regular opportunity for learning and networking; sharing the EVIPNet Europe vision and creating a joint EVIPNet Europe identity; and efforts to foster both horizontal and vertical Network growth. Additional strengths include individual motivation and personal skills at the Secretariat and country levels and the fact that there are many strong and committed national champions in the Region. This is a reflection, we believe, of the passion of people that work in the evidence-to-policy field.

A possible limitation of the EVIPNet approach is the fact that it is not linked to a specific health issue or topic. This may be limiting resource mobilisation and investment in the approach due to the lack of strong interest groups (as would likely exist for particular disease areas). It may also affect and make it more difficult to establish KTPs and embed them in local institutions.

As such, new innovative mechanisms have been explored such as establishing strategic partnerships between the Secretariat and specific health issues (e.g. antimicrobial resistance) to leverage funds for the EVIPNet Europe KT work through the country cohort approach and use of biennial collaborative agreements.

### Next steps

Areas where further attention and work are needed were also identified by the evaluation. These include the need for more sharing of lessons learnt and good practices; further expanding the capacity-building work and portfolio of instruments; and exploring the feasibility of a Russian hub/Russian coordinator for EVIPNet Europe to provide more targeted and adapted assistance to the eastern part of the Region. At the WHO Secretariat level, work is required to ensure that the staff posts remain funded. Increased funds may also be needed for further staffing in the Secretariat to support growth of the Network. Furthermore, additional Global EVIPNet capacity is needed to join up and complement regional programmes and to provide capacity-building at the WHO headquarters level.

At WHO country office level, EVIPNet Europe should continue to build capacity for EIP and clearly demonstrate and advocate for links to strategic levers, including how EVIPNet Europe can, and has, helped to achieve national health targets. At the country level, it will be important to finance and support the work of EVIPNet, ensuring that, beyond individual champions, a dedicated institutionalised, multidisciplinary EVIPNet Europe country team is created, acting as the EIP focal point to the Ministry of Health with protected time to engage in the planning, implementation and M&E of EIP work on a sustainable long-term basis [28, 60–62].

### Strengths and limitations of the evaluation

This is the first evaluation of EVIPNet Europe as a KT network of networks at its different levels (both regional and country levels). It is also one of the first, if not the first, evaluation of an organisational KT activity that is designed to improve the general climate for linking research to policy and to institutionalise EIP in countries through the creation of KTPs. Previous evaluations of EVIPNet activities have focused on specific activities, such as the evidence briefs for policy and deliberative dialogues [29], or on the impact of specific EVIPNet KTPs [27, 28, 39, 61, 67, 68]. The only known evaluations of the Network as a whole, either at regional or global level, have remained as grey literature and have not been published in the peer-review literature [69, 70]. None have explicitly included the EVIPNet Secretariat (either global or regional) as a subject in the evaluation. This evaluation of EVIPNet Europe was made even more complex by EVIPNet’s use of a systems approach to better link policy and research [63, 71–74].

The use of a peer-reviewed M&E framework, including a theory of change and logic models, is a clear strength of this evaluation. The mix of evaluation methods, including validated tools at the country level [46–50], allowed the triangulation of a wide range of data and the combination of quantitative and qualitative data, which allowed a better understanding of why things were working or not. The evaluation steering group also provided important quality assurance mechanisms.

The lack of baseline measures and a comparison group were limitations of the evaluation. However, this evaluation can now serve as a baseline for future evaluations, which could also compare changes in the use of research evidence in policy-making between more established or newly developed country teams and new EVIPNet Europe member countries. Another limitation was the possible perceived lack of independence of the evaluator. Although the evaluation was conducted by an external consultant not involved in the development, strategic thinking or delivery of EVIPNet Europe (and therefore more impartial) they could have been viewed as an internal WHO staff member by many of those who were interviewed due to the use of a WHO email address and job title. Furthermore, the response rate for the online evaluation was low (57.5%). While this response rate is higher than similar evaluations of networks [75], the findings may not be generalisable to the whole Network as it is likely that those who consider themselves progressing well were the ones who responded and those with less to report struggled. As others have suggested, generalised surveys like these for Network members should not be the dominant form of data collection [76], which is why a mixed methods evaluation was conducted. Finally, the evaluation was conducted in English and the two countries studied in depth do not have English as their first language. However, all of the people involved do have international experience, including giving presentations in English and in writing scientific papers in English; their command of the language is evidently ample.

## Conclusions

At 6 years since the launch of EVIPNet Europe at the end of 2012, the evaluation showed that the Network is valued and is filling a niche in Europe, as evidenced by the strong and rapid growth of the Network from 13 member countries to 21 in 2018 and more wanting to join. There have been some successes, including examples of KT activities influencing policy. However, no formalised KTPs have yet been created. More work and support are needed if the Network is to achieve its vision of a Europe in which high-quality, context-sensitive evidence routinely informs health decision-making processes that ultimately serve to strengthen health outcomes across the Region.

This evaluation of EVIPNet Europe as a KT network of networks that was designed to improve the general climate for linking research to policy is believed to be an important contribution to the literature. It is hoped that this contribution to the evidence base will benefit others involved in a similar process by sharing of the lessons learnt. However, EVIPNet Europe needs to build on this evaluation and regularly undertake follow-up Network evaluations. Evaluations at the country level by country teams are also needed with the support of EVIPNet Europe through capacity-building and making use of the EVIPNet Europe M&E framework. Finally, we would like to encourage other similar initiatives, including other WHO regions, to undertake similar evaluations.

## Supplementary information

**Additional file 1.** Evaluation tools.

**Additional file 2.** Detailed results for each data collection method.

**Additional file 3.** Case studies.

**Additional file 4.** Findings against the evaluation questions.

## Data Availability

Datasets used during the current study are available from the corresponding author on reasonable request.

## Abbreviations

BCA: biennial collaborative agreement
EIP: evidence-informed policy-making
EVIPNet: Evidence-Informed Policy Network
KII: key informant interview
KT: knowledge translation
KTP: knowledge translation platform
M&E: monitoring & evaluation
SAGE: Staff Assessment of enGagement with Evidence
SEER: Seeking, Engaging with and Evaluating Research
