# Supplementary material for: Evaluation of the performance and achievements of the WHO Evidence-informed Policy Network (EVIPNet) Europe
Source: Health Res Policy Syst. 2020 Sep 24;18:109. doi: 10.1186/s12961-020-00612-x (PMC7513318; doi:10.1186/s12961-020-00612-x)
Supplement: Supplementary file 1 — Additional file 1. Evaluation tools. [file 12961_2020_612_MOESM1_ESM.docx]

## Additional File 1. Evaluation tools

## A. EVIPNet Europe country online evaluation

**Country information**

What is your current role in EVIPNet Europe? [national champion OR WHO Country Office staff]

When did you start in this role with EVIPNet Europe for your country? [2013, 2014, 2015, 2016, 2017, 2018]

Have you been involved with EVIPNet Europe or evidence-informed policy-making (EIP) in a previous role? [YES/NO]

When did your country become a member of EVIPNet Europe? [2013, 2014, 2015, 2016, 2017, 2018]

Is your country one of the EVIPNet Europe pilot countries? [Yes/No]

**Evaluation**

1. 1a. In your view, has your country benefited so far from being part of EVIPNet Europe? [YES/NO]

1bi. [IF YES] please describe and give examples of how your country has benefited from being part of EVIPNet Europe. [free text]

1bii. [IF NO] please explain why you think your country has not benefited from being part of EVIPNet Europe yet (including if you have recently joined the network). [free text]

1. a. To what extent has being a member of EVIPNet Europe increased the skills and changed knowledge, attitudes and behaviours towards using evidence in policy-making of stakeholders in your country? (Please describe any difference in EIP knowledge and skills from being part of EVIPNet Europe and what you think has contributed to this). [free text]

b. Please explain what you think is required to further increase skills, and change knowledge, attitudes and behaviours of policy-making stakeholders. [free text]

1. a. Has EVIPNet Europe increased the interactions and exchange between policy-makers, researchers and stakeholders in your country? [YES/NO]

bi. [IF YES] Please provide examples of these interactions and exchanges, and explain the reasons for this increase. [free text]

bii. [IF NO] Please explain why you think this has not happened yet (including if you have recently joined the Network). [free text]

1. To what extent has being a member of EVIPNet Europe increased the awareness of and commitment to evidence in policy-making in your country? How has this been manifested? (if you are a new member please describe the current situation in your country) [free text]
2. 5a. What human resources are invested in the work of EVIPNet Europe by your country? [free text]

5b. What financial resources are invested in the work of EVIPNet Europe by your country? [free text]

5c. What time and skills resources are invested in the work of EVIPNet Europe by your country? [free text]

1. 6a. Which of the following EIP documents have you published or been working on? (please tick all those you have published or been working on)

- Situation analysis
- Knowledge translation platform (KTP) operation plan
- Evidence brief for policy (EBP)
- Policy dialogue
- Rapid response
- Monitoring and evaluation (M&E) plan

6b. [IF YES to any of above] How have/will these EIP documents be used in influencing changes in policy in your country? (Please give examples of how these documents have been used and any barriers or enablers to their development and use). [free text]

1. a. Do you learn from or do you mentor other EVIPNet Europe member countries? [YES/NO]

7bi. [IF YES] Please comment on how you do this and how useful you find it to receive and/or share lessons). [free text]

7bii. [IF NO] Do you think this would be useful and what do you think is needed to support this? [free text]

1. What would be required to develop or further strengthen KT and EIP in your country? (Please reflect on internal factors at national, organizational and individual levels, including your own role, as well as external factors, including the support provided by the WHO Secretariat and the Network). [free text]
2. a. Has your country produced any EVIPNet Europe-related peer-reviewed publications to date or are any due for publication? [YES/NO]

9b. [IF YES] Please give details of when and where these were/will be published). [free text]

Thank you for completing this evaluation.

## B. EVIPNet Europe key informant interview schedules

**Country team-level questions** *(interviewer prompts)*

1. What have been the key successes/achievements of your work/the work of the EVIPNet Europe country team to date? What have been the key barriers and challenges? *(these can be big or small)*
2. Do you think EVIPNet approaches are of value and relevance to your country? *(What makes you think this? Do you have examples of stakeholders acknowledging KT and EIP as important? To what extent do you/does* *the EVIPNet Europe country team* *approach the WHO Secretariat for advice and support? Is the value of EIP recognized by the Ministry of Health and other senior staff at country level? Are KT and EIP acknowledged as important and incorporated into stakeholders’ agendas? Are you aware of any similar EIP networks in Europe? Are you part of them? How do you they differ? What niche do you think EVIPNet Europe addresses? Do you think there is added value of EVIPNet Europe, and if yes, what is the added value?)*
3. To what extent have the activities of the WHO Secretariat of EVIPNet Europe increased the skills, knowledge, and changed attitudes and behaviours to use evidence in policy-making among the national champion(s) and/or the EVIPNet Europe country team? *(What training events took place organized by the WHO Secretariat, how were these received/attended, what was the most useful about them, what else is needed? What support was provided/was this useful [apart from training workshops, e.g. technical assistance and advice, coaching, provision of tools]? What future sessions are planned or do you think are needed? What changes have occurred as a result? What makes you think there have been changes?)*
4. How have the KT products (e.g. EBPs and PDs) been used by your country to inform policy? *(e.g.* *Do you have any examples, how do you think you will use them if you have not already, how complete, context appropriate, useful and user-friendly are the EVIPNet Europe’s portfolio of EIP tools? To what extent have they influenced changes in policy? What makes you think that? What worked well as part of the SA/EBP/PD processes? What could have worked better or been done differently?)*
5. How engaged are leaders and champions to advocate for KT/EIP in your country? *(How easy/difficult has it been to engage them? What were the main barriers/facilitators?* *Are EVIPNet Europe resources and expertise sought, discussed and utilized by external stakeholders? Do you have any examples? Is EVIPNet Europe promoted and advocated for by these leaders and champions or the country team? To what extent do you think the EVIPNet Europe national champion(s)/country team has functioned as multipliers and increased capacity and capability among national stakeholders to use evidence in policy-making? [What training events took place, organized by the national champion(s)/country team? Can you give examples, e.g. meetings/conferences where EVIPNet Europe was mentioned or the Action Plan was discussed?])*
6. What lessons have you learnt about strengthening KT/EIP and starting to develop a KTP that could be applied in other countries? *(What would you recommend repeating/maintaining or doing differently if you were to start again? What were the most challenging aspects of strengthening KT/EIP so far and setting up your KTP?* *And what have been the enablers?)*
7. How do you communicate and interact with other members of EVIPNet Europe and the WHO Secretariat for EVIPNet Europe? *(How do you interact with other member*  countries*? What information do you consider important to be shared by Network members/the Secretariat, and what information would you like to see shared more often? What have you learnt from other member countries? How have you used this? For example, have you used it in planning? Have you been able to mentor other member countries? And if so, how? What do you think would further facilitate interaction between EVIPNet Europe member countries? How does the Secretariat interact with you? Is this effective and timely? What could be improved? How do you communicate with the Secretariat?)*
8. In your opinion, what has been the most significant change(s) that took place in your country in terms of KT/EIP since your country joined EVIPNet Europe? *(this can be positive or negative)*
9. To what extent are the KT and EIP activities in your country sustainable? *(What level of support is still required from the WHO Secretariat of EVIPNet Europe? How long do you think this will be required? What steps need to be taken? What plans are in place (staffing/funding)? What factors have facilitated or hindered an increase in country KT/EIP capacity and progress towards sustainability?)*
10. Thank you for your time, that’s the end of the questions. Do you have anything further you would like to add about EVIPNet Europe? Is there anyone else you think I should interview as part of this evaluation?

**Secretariat-level questions** *(interviewer prompts)*

1. To what extent do you think the activities of the WHO Secretariat of EVIPNet Europe have increased the skills, knowledge, attitudes and behaviours among country team members to use evidence in policy-making? *(What training events took place, how were these received/attended, what was the most useful about them, what else is needed? What changes occurred as a result? What makes you think there have been changes? What were the main challenges/barriers you experienced in undertaking these activities?)*
2. How have EVIPNet Europe’s portfolio of EIP tools (e.g. EBPs and PDs) been used by country teams? *(Give examples of tools that have been used by countries; to what extent have they influenced changes in policy? What have been the barriers or enablers to developing these with countries?* *How complete, context appropriate, useful and user-friendly are the EVIPNet Europe’s portfolio of EIP tools? What makes you think that?)*
3. To what extent are the activities in EVIPNet Europe member countries sustainable? (*What level of support is still required from the WHO Secretariat of EVIPNet Europe? What factors have been/do you think will be barriers/enablers to the creation of stable KTPs?)*
4. How engaged are EVIPNet Europe members (EVIPNet Europe teams and stakeholders)? *(How many leaders and champions are engaged to advocate for EIP within countries? How easy/difficult has it been to engage them? What were the main barriers/facilitators? What makes you think they are engaged? Are EVIPNet Europe resources and expertise sought, discussed and utilized by external stakeholders?)*
5. To what extent do Network member countries promote and advocate EVIPNet Europe approaches to EIP throughout the WHO European Region? *(Can you give examples, e.g. meetings/conferences where EVIPNet was mentioned or the Action Plan was discussed? What do you think are the challenges or facilitators for member countries to do this?)*
6. What resources (human, financial, time and skills) are invested in the work of EVIPNet Europe by the WHO Secretariat of EVIPNet Europe? *(How are these invested, and how have they varied over time?)*
7. Do Network member countries think EVIPNet approaches to EIP are of value and relevance? *(What makes you think this? Do you have examples of stakeholders acknowledging KT and EIP as important? To what extent do member countries approach the EVIPNet Europe Secretariat for advice and support?)*
8. What lessons have you learnt about strengthening KT/EIP and starting to develop a KTP network? *(What would you repeat/do differently if you were to start EVIPNet Europe again? What were the main barriers/enablers?)*
9. How do you communicate with member countries of EVIPNet Europe? *(How do you share information? How do they communicate with you? How do they communicate with each other and share learning? Has this learning stimulated any changes? Can you give examples?)*
10. What accountability mechanisms for EVIPNet Europe (Secretariat and member countries) exist to measure the progress of EIP? *(How are these used/to what extent? How could they be strengthened? What are the main challenges?)*
11. Thank you for your time, that’s the end of the questions. Do you have anything further you would like to add about EVIPNet Europe? Is there anyone else you think I should interview as part of this evaluation?
