# Supplementary material for: Evaluation of the performance and achievements of the WHO Evidence-informed Policy Network (EVIPNet) Europe
Source: Health Res Policy Syst. 2020 Sep 24;18:109. doi: 10.1186/s12961-020-00612-x (PMC7513318; doi:10.1186/s12961-020-00612-x)
Supplement: Supplementary file 2 — Additional file 2. Detailed results for each data collection method. [file 12961_2020_612_MOESM2_ESM.docx]

**Additional File 2. Detailed results for each data collection method**

## A. Document review

Forty-two documents were identified and used as part of the documentary analysis (20 internal, 22 external) to provide contextual background for the evaluation and to allow further understanding of the activities conducted by the WHO Secretariat of the Evidence-informed Policy Network (EVIPNet) Europe and country teams. The documents included multicountry meeting reports (2013–2017), EVIPNet Europe annual reports (2014–2017), EVIPNet Europe tools and country products, peer-reviewed publications, capacity-building evaluation reports, WHO Secretariat and country operational plans and reports, and financial information.

## B. Social media analysis

A total of 132 people are currently members of the EVIPNet Europe Yammer group from 19 countries (N.B a number of members may have joined in previous years and since left the Yammer group and therefore would not be included in the analysis of number of registered members but may appear in the active members analysis). The largest group of members are those from country teams (51/132, 39% of members) and 20 (15% of members) are part of the WHO Secretariat (Table 1). The number of members varies by country (median 4, range 0–9). Out of all members, 48 individuals (35%) have been active over the four years since the group was created (either starting a post or commenting on a post). The most active group is the WHO Secretariat. The least active groups are the EVIPNet Europe steering group members and the WHO country office staff, with zero and two unique active members, respectively.

The most active year was 2016, in terms of new members joining, posts started, comments and “likes” received on posts, documents shared and replies to posts (Tables 1 and 2). Over the four years, the largest proportion of posts has been those of a general nature (128/359, 36%) (Fig. 1). Although this was particularly high during the first year of development, the types of post were more varied in subsequent years.

Fig. 1. Type of post by members of EVIPNet Europe Yammer all-network group, by year

Table 1. Members and activity of EVIPNet Europe Yammer all-network group, by user group and year

| **Year** | **No. of new members** | **All members** | | **WHO Secretariat** | | **Country team members** | | **Country office members** | | **EVIPNet steering group members** | | **National champion members** | | **Expert members** | |
| --- | --- | --- | --- | --- | --- | --- | --- | --- | --- | --- | --- | --- | --- | --- | --- |
|  |  | **Total no.** | **No. active^a^** | **Total no.** | **No. active^a^** | **Total no.** | **No. active^a^** | **Total no.** | **No. active^a^** | **Total no.** | **No. active^a^** | **Total no.** | **No. active^a^** | **Total no.** | **No. active^a^** |
| 2015 | 64 | 64 | 19 | 8 | 10 | 22 | 1 | 17 | 1 | 7 | 0 | 5 | 5 | 5 | 2 |
| 2016 | 38 | 103 | 27 | 13 | 6 | 38 | 10 | 20 | 1 | 8 | 0 | 11 | 9 | 12 | 1 |
| 2017 | 23 | 125 | 16 | 7 | 7 | 49 | 1 | 22 | 0 | 8 | 0 | 15 | 7 | 14 | 1 |
| 2018^b^ | 7 | 132 | 10 | 20 | 4 | 51 | 1 | 22 | 0 | 8 | 0 | 16 | 4 | 15 | 1 |
| **TOTAL** | **132** | **132** | **48** | **20** | **19** | **51** | **11** | **22** | **2** | **8** | **0** | **16** | **11** | **15** | **5** |

^a^ unique active members (Registered at that time, may no longer be a current registered member)

^b^ represents data from 01/01/2018–13/07/2018; all other years include a full year of data. This total figure for members in 2018 does not include those who joined in previous years but are no longer registered members.

WHO: World Health Organization

Table 2. Post activity of members of EVIPNet Europe Yammer all-network group, by year

| **Year** | **No. of posts started^a^** | **No. of comments received on posts^b^** | **No. of “likes” on posts** | **No. of documents shared** |
| --- | --- | --- | --- | --- |
| 2015 | 135 | 86 | 340 | 18 |
| 2016 | 136 | 90 | 335 | 102 |
| 2017 | 76 | 43 | 166 | 55 |
| 2018 | 13 | 12 | 60 | 3 |
| **TOTAL** | **359** | **231** | **901** | **178** |

^a^ Does not include groups created

^b^ Does not include comments made by the author of the post

## C. Online country evaluation

The online evaluation was completed by 23 participants (57.5% of those invited), with five of these responses only partially complete. Twelve responses were from participants from pilot countries, which are those countries that were part of the initial cohort in 2012. Eight pilot countries were selected based on a call for applications. The year during which countries joined EVIPNet Europe ranged from 2012 to 2018. Of those who responded, 12 had been involved with EVIPNet Europe or evidence-informed policy-making (EIP) in a previous role (5/12 WHO country office staff, 7/11 national champions). Given the small numbers in each group of respondents, the main results are presented in an aggregated form.

Most respondents (18/23, 78%) believed that their country had benefited from being part of EVIPNet Europe to date (Fig. 2). However, there was a mixed response to whether there had been an increase in interactions between policy-makers, stakeholders and researchers. Ten respondents (43%) reported having published or soon to be publishing EVIPNet-related work. The most commonly reported type of documents that had been published or were being worked on by countries were the situation analyses (16/23, 70% of countries) and evidence briefs for policy (14/23, 61%) (Fig. 3). The least reported type of publications were the monitoring and evaluation plan and rapid response, with only four countries having worked on each of these.

Fig. 2. Online evaluation responses

Fig. 3. Types of evidence-informed policy-making documents published or being worked on by country teams

KTP: knowledge translation platform; M&E: monitoring and evaluation

A number of themes were identified from analysing the qualitative evaluation responses to the online evaluation, i.e. questions 2, 4, 5 and 8 (Fig. 4). These are presented together for both national champions and WHO country office staff as many concepts were common to both. There were, however, more barriers/challenges identified by WHO country office staff, with more enablers/good practices identified by national champions.

Fig. 4. Key themes identified on analysis of qualitative responses to the online evaluation

**Barriers**

Some countries reported coordination between agencies to be a barrier. For example, interaction between policy-makers and the research community was lacking, there was slow coordination between ministries, or the Ministry of Health lacking a strong voice as well as interaction with the WHO Country Office. The lack of obligatory requirement or mandate for developing evidence briefs was felt to be a challenge for some countries, and the need for a ministerial order suggested.

Staff turnover was mentioned by a few respondents as causing delays in the creation of tools and institutionalization of the process, with decision-maker/government changes the main ones cited. The political will of these policy-makers was also seen as important and lack of commitment a main barrier:

*"SA is a must before we can move further but it also needs the support and will of higher-level policy-makers and researchers to make changes in the existing system and behaviours.”*

[EVIPNet Europe National Champion]

Increased awareness and engagement were mentioned, with suggestions that further promotion among stakeholders was required to support this commitment. Respondents also highlighted the importance of context and some of the challenges experienced in different countries. These included the lack of available research evidence within countries, or lack of awareness of sources, the attitudes of policy-makers and the lack of a culture of evaluation. Further issues highlighted related to the lack of appropriate translation for Russian-speaking countries:

*“The locally used terminology, even if WHO has translations in Russian using the same words, does not necessarily have the same meaning for WHO and national partners.”*

[WHO County office staff]

Capacity and resources were mentioned by most respondents as barriers. When asked about the resources invested ­– human, time, skills and financial – some were not aware of these details. Of those who were, most suggested that resources were limited, with work done on a voluntary basis, small staff numbers or contracted staff for a specific activity. Almost all finances to support EVIPNet were from WHO. There were suggestions that these current levels of funding and staffing are insufficient to sustain activity.

Institutionalization of a knowledge translation platform (KTP) was currently lacking but seen as needed by many respondents in order to systematize the process and reduce some of the impacts of turnover and lack of resources highlighted.

*“KTP institutionalization, along with stronger high-level support and involvement, would be an important way forward.”*

[EVIPNet Europe National Champion]

**Enablers**

Capacity-building support and the EVIPNet tools were both widely mentioned by both national champions and WHO country office staff and clearly valued, with examples from some on how these had, or hopefully would, change policy. However, these are currently benefiting only those who attend sessions and/or produce tools, with the recognition that ideally both the technical knowledge and practical experience are needed. Some attempts were mentioned of cascading this knowledge but there was still a need identified to widen the pool of EIP experts within countries. Mostly it was changes in skills and knowledge that were reported, although some shifts in attitudes were suggested, particularly from the policy dialogues bringing different people together to understand each other.

Sharing and networking was mentioned by several respondents, with learning lessons and exchanging experiences between countries cited as an important part of multicountry meetings, country cohorts, Yammer and direct country contact. More opportunities for mentorship and participation in international events were suggested to enhance this further.

*“It also is a good networking platform and it supports us when I see that other WHO offices are struggling with the same difficulties and see how they have solved them, etc.”*

[EVIPNet Europe National Champion]

Using strategic levers and advocacy to gather commitment was felt to be an enabler for developing EVIPNet further, such as further discussion at high-level regional policies and meetings. Others also suggested moving beyond solely focusing on health to embrace the multisectoral nature of many health policy areas and explore relationships with other ministries and sectors where these policy-makers have a role to play.

## D. Key informant interviews

Sixteen key informant interviews (KIIs) were conducted, including eight at WHO Secretariat level (three current or former WHO EVIPNet Europe Secretariat staff, three stakeholders and two from the EVIPNet Europe steering group) and eight with stakeholders from two EVIPNet Europe country teams. Data saturation was reached within this number of interviews. Interviews ranged in duration from 30 minutes to 1 hour and 6 minutes. At the WHO Secretariat level, 0% of the interviewees were male, while only 12.5% of the country team interviewees were male.

A number of barriers, challenges, enablers and opportunities were identified from the KIIs, some of which were similar to those found in the online evaluation responses (Table 3). The main themes identified were: demonstrating value; network growth; network processes; and people. Communication was a cross-cutting theme in all the interviews (Fig. 5). A brief description of each of these themes is presented below.

Table 3. Barriers, enablers, challenges and opportunities identified from key informant interviews

| **STRENGTHS**   - Strong national champion and WHO County Office engagement with EVIPNet Europe and within countries - Individual’s motivation and personal skills at the Secretariat and country levels - Multicountry meetings providing a regular opportunity for learning and networking - Technical assistance and expert support provided by the Secretariat and its partners - Training provided by the Secretariat and its partners - EVIPNet teams built within countries - EVIPNet Secretariat supports instruments and development of country KT products - WHO credibility adding value to EVIPNet’s work - EVIPNet Secretariat’s core dedicated staffing funded to coordinate and support the Network - BCA funds provide finance and contracting mechanisms for EVIPNet Europe country-level work - Development of EVIPNet Europe strategy - EIP Action Plan, adopted by all 53 Member States of WHO Europe, reinforcing the mandate of EVIPNet Europe | **WEAKNESSES**   - Complexity and added value of EIP are challenging to articulate and market - Accountability mechanisms not clear - Activity-based/mainly vertical communication between the Secretariat and Network members, while horizonal communication is still in its infancy - Human resources inadequate to undertake work at Secretariat and country levels - Literature not available (in countries and internationally) on specific topics - Political buy-in varies and is sometimes lacking, dependent on priorities - Processes of EVIPNet can be perceived as rigid - Limited time available to invest in promoting EIP which is required - Motivation and engagement varies between individuals and over time - Links to WHO strategies and policies not particularly evident at country level |
| --- | --- |
| **OPPORTUNITIES**   - Champions, with increased EIP knowledge and skills, available at country level - Career development for national champions - Country examples and success stories available and shared - Country cohort model allowing for the establishment of communities of practices - Intercountry communication and mentoring possible - Strategic partnerships with other technical areas of WHO and EVIPNet Europe - Engagement of younger generations in EVIPNet Europe - Yammer online platform opening up virtual space for network sharing and learning - Strengthening of multistakeholder relationships within countries | **THREATS**   - Financial resources lacking for Secretariat and Network member countries - Frequent turnover of staff (Secretariat and country teams) - Behaviour change is a long-term process, difficult to measure - Heterogeneous network (country teams are at various levels of implementing EVIPNet Europe activities) - Institutionalizing KTP is a complex, long-term process - Network growth leads to greater demand on scarce resources - Training in countries requires commitment from and capacity of country teams, which may be lacking and/or variable - Policy process is influenced by factors other than evidence - Return/impact of involvement with the Network hard to demonstrate - Potential for bias towards western European countries - Lack of a global role of EVIPNet in capacity-building and global networking |

EIP: evidence-informed policy; EVIPNet: Evidence-Informed Policy Network; KTP: knowledge translation platform

Fig. 5. Main themes from the qualitative analysis of key informant interviews

**Main themes**

Demonstrating value

Many stakeholders discussed the challenges that exist in demonstrating the value from EVIPNet Europe, both in terms of an academic evaluation exercise and also in a practical sense to show added value to country stakeholders to gain buy-in and support. The need for, as well as the difficulties in, measuring impact were expressed, with several highlighting that it is not straightforward and that the inability to talk about the number of lives saved, the intangibility and abstract nature of EVIPNet compared to other programmes was challenging. EIP monitoring and evaluation does not yet seem to be well developed in Member countries, as would be expected at this stage of Network development.

With the value not visible, stakeholders suggested that stories of success from other countries are important as part of EVIPNet Europe or globally. Many suggested that the EVIPNet products (e.g. evidence briefs for policy and policy dialogues) were a good way to demonstrate the value and potential of the methodology within a country.

Network growth

The Network has grown rapidly, from 13 countries initially to 21 within a few years, and more countries are seeking to join. Stakeholders felt that the growth of the Network presents both opportunities and challenges. There are more obvious challenges, such as the need for increased resources, both human and financial, required to support an increase in the number of countries. However, many expressed that they felt this was not the current case, let alone with further growth. Many suggested that the Secretariat was operating below the necessary staffing levels, often relying on a temporary outsourced workforce of consultants, interns, fellows, etc.

There were two trains of thought from stakeholders on how to deal with this growth. One option expressed was to focus on fewer countries and do this well. Others felt that the Network should continue to expand to more countries, with the longer-standing countries providing mentoring and support to newer countries, thus freeing up some capacity from the Secretariat. Another obvious challenge expressed was that this growth had led to an increased variability among countries in terms of levels of awareness, skills and knowledge. Those who have been members since the beginning will clearly have different training and support needs to those just starting. Furthermore, with this growth, some acknowledged that it would not be feasible for the Secretariat to train all country staff and stakeholders, and more emphasis would be needed on a train-the-trainer approach to be developed and then utilized by countries.

Many acknowledged that this Network growth requires increased resources and felt a possible source for that and increased coordination was required from EVIPNet Global, including the potential for global capacity-building events. There was also a sense from stakeholders that this potential had not yet been fully realized and capacity at the global level was lacking currently. Despite the growth, no KTPs have yet been developed within EVIPNet Europe. Stakeholders also acknowledged that institutionalization was not an easy or quick process but necessary if this Network was ever to become sustainable and closer to self-sufficient.

Network process

The EVIPNet process was discussed by a number of stakeholders; some felt the approach was flexible enough and others believed it to be a little too rigid. On the one hand, stakeholders expressed understanding of the need to follow the EVIPNet process, whereas others felt that developing a culture of EIP was more important than following exact methodologies. There was a need for examples of this in practice to raise the profile and show the potential benefit before interest, and hopefully longer-term funding, would follow.

Clearly the structure brought to the EIP process by EVIPNet was recognized and valued. In terms of the tools available from the EVIPNet Europe Secretariat, some stakeholders thought there were too many already and these might be overwhelming. However, countries did report using the tools and that these were relevant and useful, although many took longer than they expected to finalize them. The policy dialogue process was mentioned by several interviewees as being particularly important in developing new relationships and increasing understanding among country stakeholders. There were also plenty of examples of further tools that could be developed. Suggestions included more support for developing a rapid response service, qualitative evidence tools and more organizational management support. One area mentioned by several stakeholders as a possible gap in the tool portfolio was more information or support for institutionalizing a KTP within countries, including writing business cases, and how to finance a KTP, ensuring available resources to bring in topic experts while retaining a core of EIP experts.

People

From many of the interviewees, there was a clear sense of the importance of individuals, personalities and relationships. This related not only to the relationship with the Secretariat, with almost all country members expressing gratitude for the support received, particularly from the coordinator, but also the relationships internally with the WHO country office and Ministry of Health. Getting the right country team together was highlighted, with a need for the team to have highly motivated individuals.

Many references were made to the challenges caused by turnover of staff in all areas: national champions, WHO country offices, ministries of health and the WHO Secretariat. While there were examples of these bringing about positive changes (e.g. shift in priorities to align to EVIPNet), this was mostly seen as a negative aspect of Network development. Stakeholders discussed how it takes time to build up relationships, knowledge, skills and awareness again, and that priorities change. Turnover at the political level also had implications for how members advocate for EVIPNet, given the need for political buy-in. However, with short terms of office, ministers want to see impact quickly, while changing policy and seeing a measurable impact takes longer.

Communication

Communication was a cross-cutting theme in all interviews. Communication between countries and with the Secretariat was mostly undertaken during the development of products and other specific country activity or during multicountry meetings, with much less dialogue in between. Most stakeholders suggested communication was largely one way from the Secretariat, pushing information out. Others felt some countries seemed less comfortable approaching the Secretariat for support, with the need to build relationships and trust highlighted. Many stakeholders stressed the importance of the face-to-face elements in establishing and building these relationships.

Participants highlighted some good examples of EVIPNet Europe countries sharing experiences and stressed the importance and need for this to be further strengthened and utilized more fully. Stakeholders also suggested that more global sharing was needed; for example, learning from more established KTPs in other areas, given the lack of KTPs in EVIPNet Europe to date. Again, there were links made to the greater need for EVIPNet Global and the role of the WHO Secretariat in this coordination and global knowledge exchange and sharing, which was felt to be currently lacking capacity.

## E. Validated tools

Validated tools

*Is research working for you (IRWFY)*

Of the four domains of the IRWFY tool (acquire, assess, adapt and apply), the two selected countries both scored themselves highest on domain one: acquiring research findings (Fig. 6). The second-highest score was for domain four: applying research. While all domains scored well, both country teams still show room for improvement as would be expected with their current stage of network development. As part of the IRWFY tool, country teams were asked to use the self-assessment to reflect on their results and identify areas that need to be addressed. Both identified that in their organizations, establishing research as a priority needed either a much, or somewhat, higher priority. Both country teams felt that decision-makers currently use research inconsistently and that they need to consider research more often in making decisions.

*Staff Assessment of enGagement with Evidence (SAGE)*

It was not possible to conduct the SAGE interview with country team B within the evaluation timescales. Country team A scored slightly higher for research engagement actions (As) than research use actions (Bs) (Fig. 7). The highest score was obtained for A1: searching for research (i.e. a comprehensive search strategy was used) and A2: types of research used (i.e. finding and using a large range of available evidence). The lowest scoring item was for B4: the organization imposed the use of research, suggesting that there was no mandate for research use or expectation that it would be used.

*Seeking, Engaging with and Evaluating Research (SEER)*

The analysis of the SEER scores for country teams A and B highlighted some areas that required further improvement. These included “Tools and systems organization has to support research use”, but particularly documented processes for how policies should be evaluated; and existing relationships or established methods for engaging with research organizations. Other areas for improvement included: collaborating on competitive research grant applications; commissioning reviews of research; and assessing the usefulness of research based on likelihood of bias. Country team A answered “yes” to all three questions on generating research (undertaking, commissioning and planning of research, analysis or evaluation). Conversely, country team B answered “no” to all of these questions. The extent of research use was also higher for country team A, but for both teams research use was highest for policy or programme development, and lowest for agenda-setting/scoping, suggesting the need for further development in this area.

Fig. 6. Is research working for you (IRWFY) scores for the two EVIPNet Europe country teams

Fig. 7. Staff Assessment of enGagement with Evidence (SAGE) scores for country team A
