# Supplementary material for: Evaluation of the performance and achievements of the WHO Evidence-informed Policy Network (EVIPNet) Europe
Source: Health Res Policy Syst. 2020 Sep 24;18:109. doi: 10.1186/s12961-020-00612-x (PMC7513318; doi:10.1186/s12961-020-00612-x)
Supplement: Supplementary file 3 — Additional file 3. Case studies. [file 12961_2020_612_MOESM3_ESM.docx]

## Appendix 3. EVIPNet Europe Country case studies

| **Country** | **Republic of Moldova** |
| --- | --- |
| **Joined EVIPNet Europe** | 2013 |
| **Key members** | |
| **Marcela Tirdea**, Head, Division of Policies Analyses, Monitoring and Evaluation, Ministry of Health, Labour and Social Protection of the Republic of Moldova  Angela Ciobanu, National Professional Officer, World Health Organization, Country Office  Galina Obreja, Associate Professor, State University of Medicine and Pharmacy | |
| **Country activity and key milestones** | |
| Since joining the Evidence-informed Policy Network (EVIPNet) Europe in 2013, the Republic of Moldova has undertaken a **situational analysis** and finalized an **evidence brief for policy (EBP)** on “Informing amendments to the alcohol control legislation” directed at reducing the harmful use of alcohol in the Republic of Moldova.  After developing the final draft of the alcohol consumption EBP, they conducted **policy dialogues** (PDs) on the same topic with key stakeholders.  Further EBPs are now under way, including on the topics of antimicrobial resistance (AMR, as part of a cohort of EVIPNet Europe countries) and reducing salt consumption. | |
| **Successes** | |
| An important achievement for Moldova was the **change in** **legislation** based on their EBP and PDs focused on alcohol consumption through amendments to the alcohol control policy. Beer was previously considered as a food, but following this work, it is now classified as an alcohol product and therefore subject to all the existing alcohol legislation within Moldova.  The EBP was also used by the Ministry of Health, Labour and Social Protection to inform and develop their action plan for reducing drunk–driving accidents.  The EVIPNet Moldova team are now trying to use their EBP to influence further legislation changes, including modifications related to price, taxes and advertising of alcohol.  *There was a change in the policy and beer, which was previously considered as a food … was accepted by the alcohol board. In 2017, it was again included under this law, I think that’s the greatest success we have achieved.*  a  The **PDs** were also a great success. Although they had previous experience in organizing PDs, the training and capacity-building workshops and activities that the Moldova team had attended as part of EVIPNet Europe led to the PD being more structured, with evidence being the most important element. The team now have both the essential theoretical knowledge and practical experience of how to develop policy and present an evidence brief within the PD. | |
| **Challenges** | |
| Achieving these successes has not been without challenges for the Moldova EVIPNet team.  One of these challenges has been the **recognition and understanding** of evidence and the need for it. While it is widely accepted within the country that all policies should be evidence based, sometimes there were misconceptions regarding what evidence really is and where to find it. The whole team used every possible opportunity to inform stakeholders about the EVIPNet Europe approach and share examples about their work, what evidence is, and how it can be found and used in policy-making. Although this took some time, this was a necessary process for stakeholders to really understand and accept the approach.  *I think first of all they really need recognition and acceptance. They accept and really recognize that all policies have to be evidence based but, at the same time, they have misconceptions about what evidence really is.*  There were also challenges in terms of **time, capacity and turnover** of staff. This was evident in all stakeholder organizations, but was felt particularly in the case of those from the Ministry where turnover necessitates re-education about the EVIPNet process and starting from scratch to develop the skills and build experience again.  *So, in the Ministry of Health, people work very little, for a short period of time and individuals change every two, three years and because of this they are not able to ensure sustainability, because not only knowledge, but also skills and experience accrued during our experimental development of the EBP on alcohol is very important and needs to be used for developing other EBPs.* | |
| **Knowledge translation capacity- and skill-building** | |
| Members of the EVIPNet Europe team in Moldova have taken advantage of a number of capacity- and skill- building opportunities made available, and then been able to use and apply this learning in practice. These include the following:   - **Country training** jointly organized with the WHO Secretariat and the EVIPNet Slovenia team. This included stakeholders from the Ministry of Health, Labour and Social Protection, the National Institute of Public Health, nongovernmental organizations (NGOs) and academia. Attendees learnt how to find, assess and use systematic reviews, and the knowledge was then used in practice when drafting their EBP. - **Multicountry meetings**   - Members attended training on how to organize a PD and then used that knowledge at country level for their PDs on alcohol consumption.   - Members also used these meetings as a great mechanism to ask questions, clarify problems, share experiences and learn from other colleagues across EVIPNet Europe.   - Members attended workshops on EVIPNet and EBPs, including how to define the problem, how to find the solution, practical application, how to make a summary, and use existing evidence for promoting the use of health evidence in the country. After that, they used the knowledge and skills in developing their own EBP and advocate for the EVIPNet approach. - **Webinars** organized by the WHO Secretariat were accessed by the team. These also provided an opportunity for other colleagues, who may not be able to attend multicountry meetings, to participate. - **HINARI training** was organized by the WHO Secretariat, which included information about the resources available that the team could use. - **Mentorship and coaching** from the Knowledge to Policy (K2P) Center in Lebanon.   *…huge difference before 2012 and now; we have started to use systematic reviews now in the policy-making process, based on the capacity-building events, training, which the Secretariat provided to Moldova.* | |
| **Knowledge translation and value and culture of evidence-informed policy-making** | |
| Over the years, the Moldova team feel that the **culture of evidence-informed policy-making** (EIP) has changed since being part of EVIPNet Europe. One reason for this is the growth in the number of people who are now engaged in and supportive of EIP and truly understand it. Even the use of the word “evidence” itself has increased from being hardly used (or used incorrectly to only refer to data) to now being used all the time.  *We have a bunch of people fully supporting this process and they understand now what is evidence and what is the need for this evidence to support policy development and so on. It changed from the point when we started to work at country level till now and yes, I can see the changes and more engagement from national counterparts in this.*  The team in Moldova put in a lot of work with the WHO Secretariat and WHO Country Office to bring this message to the leaders. It was not always easy in the beginning, and although leaders would talk about needing good policies, and using concrete evidence that demonstrates effectiveness and–cost effectiveness, it was more challenging and the use of evidence was inconsistent.  The EVIPNet team are continuing this approach, constantly trying to advocate for knowledge translation (KT) and using EBPs for all health policies developed by the Ministry of Health, Labour and Social Protection. | |
| **Lessons learnt** | |
| ***Start with the situation analysis***  The development of a situation analysis is an important part of the EVIPNet Europe process. It might seem like a lot of work and take some time but Moldova found it a key tool to provide a good grounding and knowledge about where the best place is for a knowledge translation platform (KTP). The assessment helped them understand where to find the political will, which is needed to accept and integrate the KTP.  *We started with a situation analysis and for me at least, and for my colleagues, it was very useful. We understand who are the stakeholders, what they do, what kind of communication is between them, how they work, when they share or do not share something or nothing, so for us it was very, very useful.*  ***Get the team in place***  Another very important lesson Moldova learnt was developing their EVIPNet team early on. This involved working very closely with the WHO Country Office, the EVIPNet Europe Secretariat and the Ministry of Health, Labour and Social Protection. With all partners providing mutual support, it is much more likely to be a success. Once established, good collaboration and continuous information exchange is one of the key factors in enabling continuous engagement and cooperation.  *To create this connection, a strong connection between the EVIPNet Secretariat, WHO Country Office and the team is very important. Whether there are two or three people, or even one, depends, but having dedicated people is important and works, at least for Moldova.*  ***Capacity-building***  Once the team was in place, building skills was seen as vital. This started with learning about what an EBP means, how to develop one and for what reason. It included training for the team on how to find evidence, how to apply and use it, and how to synthesize it. It is also very important to continue this capacity development and then cascade the information further to stakeholders.  *I think first of all they have to start with capacity-building and spread information as widely as possible about the importance of KTP and EBP.*  ***For policy dialogues, preparation is key***  Moldova also learnt a lot from the experience of conducting PDs. When preparing, they considered the possible scenario in advance so that they were aware of how to proceed and had thought through how to manage the session. They recommend that two people should moderate the PD as it is not an easy activity to do all day. They also believe that the invitation list is important; to bring together different people with differing views is crucial – even if you might not to be comfortable yourself! You want people who represent different views to attend the PD and really gain from their input. Having the evidence alone is not sufficient if there are people you have not invited who you need in order to develop the policy or ensure that the law is approved.  *One lesson I learnt during PD is its preparation, so the biggest and most important thing for a successful PD is preparation…. I think 70% of the success of a PD is preparation.* | |
| **What is the most significant change?** | |
| In Moldova, the most significant change is that the **quality of policies has improved**. The country now uses evidence to inform every policy, it addresses the question in a different manner and always includes how their policy will improve the health of the population, which groups of the population, and by how much. This, it believes, has come from a recognition of the EBPs; the structure and format is well received, transparent, organized and useable, leading to increased engagement of national stakeholders who now demand the production of additional EBPs using the same methodology. This has also led to **improved communication** between researchers and policy-makers using these structured processes.  *Now we know how to use evidence in providing information to our decision-makers, what is best to do to improve the health of the population. How to arrange the evidence, what to put in the first place, what to put in the second place, and how to provide this information in friendly language for decision-makers.* | |
| **Next steps** | |
| There are exciting times ahead for the Moldova EVIPNet Europe team, with **two more EBPs under way**, the one on AMR due to be published any day and the next one on salt consumption currently in development.  The team is ambitious in their desire to start delivering training within the country to wider stakeholders. It hopes to work with the Ministry of Health, Labour and Social Protection to organize additional **capacity-building** events to promote and strengthen what has already been achieved since joining EVIPNet Europe, and to move forward to train more colleagues with this knowledge and about this EIP methodology.  In the longer term, the team is making important preparations to establish a **KTP** within the country. It is working hard to be in the best possible position to capitalize on any opportune moments that occur, given the political dimensions involved in KTP establishment. It hopes to use the intersectoral collaboration required by the Sustainable Development Goals (SDGs) as a mechanism to work with other ministries and the recently expanded Ministry of Health, Labour and Social Protection further on institutionalizing EIP in Moldova. | |
| **Publications** | |
| ***Moldova EVIPNet Europe-related publications to date:***   - [A national dialogue on the evidence for the reduction of alcohol consumption in the Republic of Moldova](http://www.euro.who.int/en/countries/republic-of-moldova/news/news/2017/09/moldovan-ministry-of-health-convenes-second-policy-dialogue-on-the-evidence-for-strengthening-alcohol-control-interventions). In: WHO Regional Office for Europe [website]. 2017. - [Evidence brief for policy. Informing amendments to the alcohol control legislation directed at reducing harmful use of alcohol in the Republic of Moldova](http://www.euro.who.int/en/countries/republic-of-moldova/publications/evidence-brief-for-policy-informing-amendments-to-the-alcohol-control-legislation-directed-at-reducing-harmful-use-of-alcohol-in-the-republic-of-moldova-2019) (WHO EVIPNet Europe). Copenhagen: WHO Regional Office for Europe; 2019. - [Evidence-informed Policy Network (EVIPNet) Europe: success stories in knowledge translation](http://www.euro.who.int/__data/assets/pdf_file/0003/375078/EVIPNET.pdf). Public Health Panorama. 2018;4(2). | |

| **Country** | **Slovenia** |
| --- | --- |
| **Joined EVIPNet Europe** | 2013 |
| **Country activity and key milestones** | |
| Slovenia is one of the EVIPNet Europe pilot countries. Since officially launching in 2014 national EVIPNet Europe activities, the country, the country team has been working on **two evidence briefs for policy (EBPs):**   - Payment mechanisms of general physicians’ and family doctors’ outpatient clinics for strengthening care provision and management in Slovenia - Antibiotic prescribing in long-term care facilities for the elderly   Slovenia was also the first member of EVIPNet Europe **to publish their situational analysis in 2017,** providing a deeper understanding of the national evidence-informed policy making context. | |
| **Successes** | |
| Slovenia’s two EBPs have been used in a number of ways so far **to inform policy:**   - The Payment mechanisms of general physicians’ and family doctors’ outpatient clinics for strengthening care provision and management EBP was officially presented at an international policy dialogue on Primary health care in June 2017. The Ministry of Health highly appreciated its value and used it when preparing for drafting the new national Primary Health Care strategy. While the latter was eventually not adopted by the government, the evidence brief informed the Ministry’s discussions with the Health Insurance Institute of Slovenia, and may, furthermore, be drawn upon as an input to the development of the new Primary Health Care strategy. - The Antibiotic prescribing in long-term care facilities for the elderly EBP was developed as part of the first **cohort of EVIPNet Europe countries, working on AMR.** EBP has been incorporated into the development of Slovenia’s national action plan and strategy on AMR. Thus, in the future at least one of the three elaborated options will be actually implemented into nursing homes. And preparations are well under way for the related policy dialogue in November 2018, where they are hoping new guidelines for the administration of antibiotics in institutional care for elderly will be adopted [Update: following the policy dialogue, which took place in November 2018, the Former State Secretary of the Ministry of Health Pia Vračko public announced that all three options will be included into the revised national AMR Action Plan for further implementation].   For all the EBPs a key success outcome was also how the whole process brought together clinicians, public health specialists but also experts from other fields within Slovenia, **building relationships** which hopefully can be utilised further in the future, not just in the EVIPNet related tasks.  The EBPs have had another added benefit in developing and **strengthening awareness** with stakeholders in Slovenia about the importance of having a rigorous system of considering the evidence, both local and international in taking policy decisions.  *It is my opinion that an increasing number of people are thinking about it. So when an issue expected to lead to a new policy proposal comes up, then there are more people who instinctively think about the benefits and harms, wonder whether they have considered the evidence there is about benefits and harms of the policy proposal, its costs and the cost effectiveness, the related implementation challenges and so on and so forth. So what I’m trying to say is that the major achievement has been in this evolvement of the thinking about the policy proposals that is spreading among stakeholders, in my opinion particularly since EVIPNet was launched.* | |
| **Challenges** | |
| Whilst evidence-informed policy-making (EIP) is not a new concept in Slovenia and is recognised as important, there have also been challenges along the way for the EVIPNet team to overcome.  **Policy dialogues** are not that common, so this has been a new process for the team to go through and develop as a model to go forwards, learning along the way how to conduct the dialogue themselves rather than have others do it for them.  **EIP is recognised** as important but further down the policy making process other factors can come into play, over which you have less control. Changing culture and understanding takes time and a lot of energy, but there are signs that this is happening.  **Developing a Knowledge translation platform (KTP)** in Slovenia is also taking longer than everyone thought, although there have been many positive steps towards it.  **Capacity** is always a challenge; everyone has busy jobs and other tasks, with EVIPNet Europe work not always seen as a priority by those in higher level positions, so often this work has been done on top of the member’s day jobs. However time and dedication from the Slovenia team have helped overcome this. | |
| **Knowledge translation capacity and skill building** | |
| EVIPNet Europe members from Slovenia participated in trainings and meetings organised by the WHO Secretariat of EVIPNet Europe, including how to conduct literature searches and referencing, which were then used alongside the materials and tools as part of the WHO EVIPNet platform to get started on their EBPs.  There is promising work to train the next generation of public health professionals and those from other specialties in Slovenia about EIP through teaching by the national champions within relevant undergraduate and postgraduate curricula. Further work is required to extend the knowledge gained by the EVIPNet team to a wider range of stakeholders in Slovenia, including civil servants working across agendas, on EIP methodology using a **train the trainer model.**  *The understanding, how important is to have the evidence, and moreover how to collect it and what really is evidence. Because not everything, what is data and information is immediately evidence, so how really to produce evidence from available information and data, so this is probably the biggest step forward in the work of Slovene colleagues. For sure for decades they have been using the word evidence but I think the real substance and how to make it very strong evidence, I think they learnt a lot during these three years.* | |
| **Knowledge translation and evidence informed policy making value and culture** | |
| In Slovenia, EIP is acknowledged as important and is increasingly being recognised by people from the Ministry of Health and the National Institute for Public Health as the right methodology to work on policy preparation. For example, there is a growing understanding that there should be a structured process, including the preparation on an EBP, and that this is now recognised as the correct way to work in EIP.  *Everybody we have spoken to stakeholders, presentation of EVIPNet of the various products, that we have done, the value of what was recommended by EVIPNet has been recognised by everyone as far as I can tell. I don’t think you can find anyone in Slovenia who can see we don’t need to consider the evidence when we take policy decisions.* | |
| **Lessons learnt** | |
| The Slovenia EVIPNet Europe team have learnt much on their journey since 2013, but here are their **top four T’s for success**:   - ***Time – everything takes longer than you think!***   The KTP is still to be formally established and the EBPs and situational analysis also take time including the review and editing stages. However, this is often part of the learning and a necessary process to ensure these documents are of the high quality required. Don’t lose hope and keep each other motivated to continue pushing things forward and you will see changes.   - ***Team*** - ***is everything!***   The partnership you create is so important. In this case a strong team was created with members from the WHO Secretariat, the WHO Country Office, the Ministry of Health and the National Institute for Public Health, crucially, all those involved have an understanding and want to be involved in the process. The team for producing an EBP is just as critical, it’s very important who you engage in your working group, with a mix of content and technical experts.   - ***Topic – choose wisely!***   Selecting a topic for your first EBP is important; think about something that will be attractive to the Ministry of Health so that you can demonstrate value and gain interest. But you also need to consider if it is a viable topic with achievable outcomes. In this sense a narrower topic may be more suitable for your first EBP.   - ***Top level buy in – seek support***   Gaining high level engagement and support from the Ministry of Health is vital for the success of the EVIPNet team. Identifying a champion and demonstrating the added value of the EVIPNet approach helps to progress.  *The successes relied heavily on the support of our current director and our previous director… some people from Ministry of Health who are very supportive of EVIPNet and I think this is key and we wouldn’t have gone so far without that.* | |
| **Most significant change?** | |
| Since having joined EVIPNet Europe the most significant change that has taken place in Slovenia has been **a change in understanding**. There is now an increased awareness and understanding of what evidence really is, why it is important, and how to use it with a more structured and systematised approach.  The team believe the country now has a more rigorous process to consider policy making decisions, including the involvement of stakeholders, the consideration of harms and benefits, the cost effectiveness and tabling several options, more now than they did before being part of EVIPNet Europe.  *So it’s not just the systematisation of what has already been done but also of enriching what was being done in considering that fact you need to have a policy dialogue, considering the fact that you need to consider the cost effectiveness, thinking about maybe it’s a better approach to erm present several options. This is something that was not and I believe would not be without EVIPNet something that we would think about.* | |
| **Next steps** | |
| The EBP on antibiotic prescribing is due to be published any day, and **the policy dialogue** planned for November 2018 [Update: the EBP is available now online, see [here](http://www.euro.who.int/__data/assets/pdf_file/0004/386419/evipnet-euro-slovenia-no3-eng.pdf?ua=1), and the policy dialogue took place, see [here](http://www.euro.who.int/en/countries/slovenia/news/news/2018/11/promoting-evidence-informed-policy-development-to-combat-antimicrobial-resistance-in-slovenia)), with the team hopeful this will feed into the national AMR strategy as signs are already positive. As there will be strong participation and engagement from the Ministry of Health at the policy dialogue, the team are also helpful to capitalize on this opportunity to again discuss how to proceed in the future with sustainability of the process. **Establishing a national Knowledge Translation Platform (KTP)** is the major next step for Slovenia. Whilst this is taking longer than everyone thought, there have been many positive steps towards it, including a commitment to institutionalize by 2019.  *I think EIP will remain sustainable anyway otherwise it cannot be done in the future. The only question is if it will be diluted, you know that every group has its own evidence people, or seek them ad hoc when they need something to support the work, or whether there will be a core body with the roster of all possible experts and evidence of who can contribute with what and they would be approached in the first instance when the ministry needs something.* | |
| **Publications** | |
| ***Slovenia EVIPNet Europe related publications to date:***   - [Evidence brief for policy. Antibiotic prescribing in long-term care facilities for the elderly](http://www.euro.who.int/en/countries/slovenia/publications/antibiotic-prescribing-in-long-term-care-facilities-for-the-elderly)) *(2018)* - [Situation analysis on evidence-informed policy-making](http://www.euro.who.int/__data/assets/pdf_file/0019/367111/evipnet-euro-series-no1-eng.pdf) *(2017)* EVIPNet Europe Series, N°1 Slovenia - [Evidence-informed policymaking in Slovenia](http://www.euro.who.int/__data/assets/pdf_file/0019/312319/Eurohealth-volume22-number2-2016.pdf?ua=1) *(2016) Eurohealth, Volume 22, Number 2,* - [Case study: The evidence-informed policy-making context in Slovenia: ground work](http://www.euro.who.int/__data/assets/pdf_file/0010/317539/7-Case-study-Evidence-informed-policy-making-context-Slovenia.pdf?ua=1) (2016) Public health panorama, Volume 2, Issue 3, 249-400 | |
