# Supplementary material for: Evaluation of the performance and achievements of the WHO Evidence-informed Policy Network (EVIPNet) Europe
Source: Health Res Policy Syst. 2020 Sep 24;18:109. doi: 10.1186/s12961-020-00612-x (PMC7513318; doi:10.1186/s12961-020-00612-x)
Supplement: Supplementary file 4 — Additional file 4. Findings against the evaluation questions. [file 12961_2020_612_MOESM4_ESM.docx]

## Additional File 4. Findings against the evaluation questions

Findings from all sources are presented here under each of the evaluation questions and sub-questions (from Table 1).

EQ 1. Is EVIPNet Europe needed and accepted by member countries?

*1a. How effective is communication in both directions between the EVIPNet Europe Secretariat and the members of EVIPNet Europe?*

Communication has so far mainly been one way from the WHO Secretariat to member countries of the Evidence-informed Policy Network (EVIPNet) Europe via email and phone/Skype to disseminate information. More two-way communication has been provided through key network activities, such as the multicountry meetings and national training. Working on specific knowledge translation (KT) activities with member countries also develops two-way communication, e.g. via development of evidence briefs for policy and the recently established country cohorts working on evidence briefs addressing a similar high-priority issue (the latter being an example of cross-country communication). Network member countries reported feeling supported by the WHO Secretariat and did not raise any issues communicating with it. The potential to further cater to the communication needs of Russian-speaking Network members was raised as an area for future improvement. It was noted that the Network’s close collaboration with WHO country offices has been highly beneficial because it allows for context-sensitive approaches and a good understanding of the political processes.

In general, Yammer, with 132 registered members since its inception in 2015, has the potential to function as an effective communication platform, although time constraints and social media overload are challenges to regular use. The evaluation findings show that many stakeholders had heard of Yammer, with only a small number of EVIPNet Europe member countries not being part of the online community. The height of activity on Yammer was in 2016. Impetus and momentum seem to have decreased more recently, with the Yammer Coordinator’s post being vacant within the Secretariat.

*1b. To what extent do member countries recognize and support EVIPNet Europe’s strategies, objectives, mandate and function?*

EVIPNet Europe’s strategy, objectives and function are clearly outlined in its own strategic documents and are actively promoted by the WHO Secretariat. At a regional level, the Network’s objectives and function were highly welcomed and valued. The lack of global coordination of EVIPNet at WHO headquarters was noted, with an unmet potential of the Global Network to raise awareness and visibility.

*1c. What factors at country level have facilitated or hindered the creation of stable and sustained research-to-policy activities and environments in EVIPNet Europe member countries?*

While EVIPNet country teams that advance the planning and implementation of KT activities have been formed, knowledge translation platforms (KTPs) have not been formally established yet in any Network member country. Several member countries have produced, or are undertaking, a situation analysis supported by the WHO Secretariat and its tools, as a first step towards the creation of a KTP. However, building a KT infrastructure is a comprehensive, iterative and long-term process involving systemic changes. Some of the pioneering member countries (such as Slovenia and Hungary) are currently negotiating the establishment of such structures. Additional training and support instruments for member countries on this aspect of institutionalization would be beneficial and are being developed by the WHO Secretariat.

A number of factors were identified that have hindered the development of KTPs in member countries. These included:

- the need for political buy-in, recognized by all as an important lever for the success of EVIPNet;
- further advocacy required at country, regional and international levels to increase visibility and interest;
- the lack of clear national policies or rules and regulations that would make evidence-informed policy-making (EIP) processes mandatory or the lack of implementation of these processes;
- frequent turnover of staff in relevant organizations;
- lack of resources (human and financial);
- the complexity of KT and EIP;
- the weak coordination between agencies;
- lack of time, recognition, awareness and commitment;
- specific country contexts, e.g. availability and accessibility of evidence, language challenges, etc.

Enablers identified included:

- capacity-building by the WHO Secretariat and its partners, e.g. mechanisms such as country cohorts, sharing of examples and good practices, using EVIPNet support instruments;
- networking and relationships (nationally, regionally and globally, allowing for peer support and mentoring) and the creation of intercountry relationships;
- contracting – contracts for work undertaken by national champions and the inclusion of EVIPNet Europe in biennial collaborative agreement (BCA) mechanisms, which are contracts between the WHO Regional Office for Europe and a Ministry of Health. Official priorities for collaboration are set for a period of two years;
- using strategic levers;
- demonstrating wider impacts.

EQ 2. Is EVIPNet Europe achieving its objectives?

*2a. How actively are EVIPNet Europe members engaged with the Network and participating in its activities at WHO Secretariat and country team levels?*

A large volume of activities is conducted with, for and by participating EVIPNet Europe member countries. The level of engagement, however, varies, with some member countries and individuals clearly more engaged than others. This translates into varying levels of progress of Network members in advancing with the implementation of KT activities. For example, some member countries are on their second or third evidence brief for policy, whereas others are yet to finish their first one. This can, on the one hand, be explained by the variation in the dates of joining EVIPNet Europe, and on the other hand, by factors such as the frequent staff turnover at the country level.

*2b. To what extent does EVIPNet Europe increase effective joint working, networking, collaboration and sharing of lessons learnt between individuals and organizations at the national, regional and international levels?*

The Secretariat has put in place numerous and diverse mechanisms to develop joint working and networking.

- Yammer was seen as a useful platform for additional networking across EVIPNet Europe, with a key benefit being its use as a repository for information, reference and sharing success stories. Most respondents thought of this remote virtual platform as a complementary tool, which in itself is insufficient to keep people networking and sharing.
- The interpersonal contact of face-to-face meetings was highly valued for developing and maintaining relationships and are an important part of the Network.
- Country cohorts were highlighted as an area of innovation and good practice for continued technical assistance by the WHO Secretariat to Network members and mentoring, e.g. when a group of countries work on similar KT products such as developing an evidence brief for policy on antimicrobial resistance (AMR). These, furthermore, allow the creation of communities of practice and strengthen intercountry sharing and support. The country cohorts also allow the building of strategic partnerships in-house between the WHO Secretariat of EVIPNet Europe and technical colleagues (e.g. working in AMR), enable the securing of additional resources (e.g. the EVIPNet Europe funds are complemented by those from the AMR programme), as well as provide support and enhance senior buy-in. Many suggested that this should be replicated across WHO programme areas.

While initial lessons learnt and country success stories are being made available through the above mechanisms, the need was expressed to further identify and share lessons learnt and good practices regionally and globally.

*2c. What are the resources (human, financial, time and skills) invested into the work of EVIPNet Europe at WHO Secretariat and country team levels?*

An economic evaluation was beyond the scope of this review but, on the surface, it would appear that much is being achieved with very little input in terms of the financial and human resources assigned. However, the human resource input in terms of the actual hours worked on this is evidently higher, with much of this an add on, due to the motivation and enthusiasm of a few individuals. Staffing within the Secretariat has been at times highly volatile and reliant on short-term contracts or external support, resulting in loss of continuity and organizational memory. How sustainable this low capacity is with the growth of EVIPNet Europe in the long term is unknown, with potential negative consequences for the quality and level of support available, relationships, Network outputs and individuals

There are concerns about the impact on the growth of the Network, given that capacity and resources are currently limited at the Secretariat level. Activities are not yet sustainable at country level without WHO funding. Country teams are often reliant on a few staff doing the work over and above day jobs without dedicated funding or terms of reference. Turnover, which is to some extent inevitable, can, in this context, understandably have a big impact. This is largely due to the fact that, as of now, no formalized KTPs exist within EVIPNet Europe. Most input to date has focused on country launches, establishing smaller country teams and ad-hoc networks, and developing country KT products (including demonstrating the value and proof of concept of EVIPNet and its approach). With this growth, there is now a need for resources to also focus on the sustainability elements for more developed countries to support them in their efforts to institutionalize EIP.

*2d. To what extent have EVIPNet Europe and country teams developed skills and changed knowledge, attitudes and behaviours to use evidence in policy-making?*

There is evidence that EVIPNet’s capacity-building programme of work is bringing about changes in knowledge and skills at individual levels for those who attend training and engage with the Network. Although attitudes and behaviours are harder to quantify, there are signs that these too have improved: individuals are now more aware, confident and have practical experience in finding, appraising and using research evidence, as demonstrated by the activities and products developed by countries. There is limited evidence that this change in behaviour has been achieved, beyond the individual level, at an institution or country level, largely due to lack of capacity, processes and time available for EIP. Capacity-building and behaviour change are, in general, time-intensive exercises, even more so at the institution or country level, which require system-level changes.

The before-mentioned Network growth also impacts on the required level of training and support provided by the WHO Secretariat. New Network member countries have needs that are different from the more mature ones. Growth therefore presents challenges to providing tailored training from a resource point of view, since the portfolio of training activities needs to be expanded.

EQ 3. What are the outcomes of EVIPNet Europe?

*3a. What accountability mechanisms are in place for EVIPNet Europe (Secretariat and member countries) to measure the progress of EIP?*

Many stakeholders were not aware of the accountability mechanisms that exist for EVIPNet Europe. There was widespread understanding that monitoring and evaluation were an important but challenging aspect of providing accountability. Contractual elements with member countries were highlighted as an accountability mechanism, with further use of the BCAs encouraged. There is possibly a need for more transparency of the accountability mechanisms that exist, e.g. what and how the WHO Secretariat and member countries report, and to whom.

*3b. To what extent has EVIPNet Europe’s portfolio of EIP tools been used by country teams to influence changes in policy?*

The main measurable outputs at this stage of Network development are the Secretariat support instruments, which were valued and perceived as useful, and the country activities/products developed using these. A number of EVIPNet Europe member countries have successfully produced and published outputs such as evidence briefs for policy and organized policy dialogues, which are achievements in themselves. Indeed, other EVIPNet Global member countries have also used these KT tools, which were well received and viewed favourably [1], as they have been in EVIPNet Europe.

There have been some examples of KT activities influencing policy, e.g. Estonia’s evidence brief for policy on sugar-sweetened beverages provided the basis for the Parliament to propose the introduction of a tax on non-alcoholic, sweetened beverages, and the Republic of Moldova’s evidence brief for policy on alcohol consumption influenced the alcohol control legislation: beer, which was previously categorized as food, is now legally recognized as an alcohol product. While it is challenging to attribute cause and effect, countries felt that the EVIPNet approach and activities played a large part in these changes. These KT activities need to be scaled up. Policy dialogues, organized following pre-circulated evidence briefs, were highlighted as a promising way to influence policy and the wider EIP culture at a country level. Policy dialogues develop an awareness and understanding of EIP and are a good way of demonstrating the value of the process at a senior level.

*3c. Does EVIPNet Europe fill a niche in the European health policy environment?*

EVIPNet appears to be filling a niche in the Region, with its focus on developing KT networks, teaching countries and building capacity. There is an expressed demand for EVIPNet Europe, as demonstrated by the rapid growth since its inception. Originally starting with 13 countries in 2012, it grew to 19 in 2016 and 21 in 2018. Informal requests from additional countries wanting to join suggest that there is a continued demand for the Network.

*3d. To what extent do Network member countries value, promote and advocate for EVIPNet Europe’s function and approaches throughout the WHO European Region?*

The evaluation suggests that the Network is valued by its members, who serve as advocates for (i) the Network’s approach in terms of creating a higher commitment to EIP at a country level and the need for systematic and transparent processes for accessing, appraising, synthesizing, translating and applying evidence, and (ii) the use of the support instruments. It was suggested that the portfolio and order of activities should remain flexible to allow adaptation to country contexts.

The need for EVIPNet has, for instance, been expressed and promoted by countries at high-level meetings (e.g. at Regional Committee and country meetings), demonstrating interest and value, and that it is filling a current gap in Europe. Moreover, through publications, conference presentations, and the organization of national conferences and workshops by EVIPNet Europe member countries, this advocacy is starting to reach a wider audience. Given the variation in country engagement in the Network, there is also variation in the number of promotion activities, such as presentations, publications and success stories shared, with the pioneer countries being the most visible throughout the Region.

*3e. How have the Network relationships, contacts and exchanges created by EVIPNet Europe influenced the country EIP environment?*

At a regional level, the EVIPNet Europe Secretariat has built a network structure and used a variety of mechanisms to enhance relationships and exchanges between countries. As mentioned earlier,

country cohorts were referenced as a promising model, particularly the community-of-practice element. Multicountry meetings and Yammer are also useful mechanisms for building relationships. The extent to which these relationships have led to changes in the country-level EIP culture is challenging to quantify, but they have influenced processes and development of KT activities in the countries that have reached out.

At a higher level, the WHO Europe EIP Action Plan [2] is a key document in changing culture. Exposure to, and recognition by, Member States of its importance has been demonstrated, which paves the way for EVIPNet Europe to be developed as an integral aspect of EIP within countries. There is recognition that changing the EIP environment at a country level requires long-term commitment and therefore it is to be expected that these outcomes have not yet been fully achieved, but promising steps have been taken.

**References**

1. Moat KA, Lavis JN, Clancy SJ, El-Jardali F, Pantoja T, Knowledge Translation Platform Evaluation study team. Evidence briefs and deliberative dialogues: perceptions and intentions to act on what was learnt. Bull World Health Organ. 2014;92:20–8.

2. WHO Regional Office for Europe. EUR/RC66/12 Action plan to strengthen the use of evidence, information and research for policy-making in the WHO European Region. Copenhagen: WHO Regional Office for Europe; 2016.
